# Identification of New L-Fucosyl and L-Galactosyl Amides as Glycomimetic Ligands of TNF Lectin Domain of BC2L-C from *Burkholderia cenocepacia*

**DOI:** 10.3390/molecules28031494

**Published:** 2023-02-03

**Authors:** Sarah Mazzotta, Giulia Antonini, Francesca Vasile, Emilie Gillon, Jon Lundstrøm, Annabelle Varrot, Laura Belvisi, Anna Bernardi

**Affiliations:** 1 Dipartimento di Chimica, Università degli Studi di Milano, 20133 Milan, Italy; 2 Université Grenoble Alpes, CNRS, CERMAV, 38000 Grenoble, France

**Keywords:** lectins, fucosides, *N*-glycosides, crystallography, glycomimetics, antiadhesive therapy

## Abstract

The inhibition of carbohydrate-lectin interactions is being explored as an efficient approach to anti adhesion therapy and biofilm destabilization, two alternative antimicrobial strategies that are being explored against resistant pathogens. BC2L-C is a new type of lectin from *Burkholderia cenocepacia* that binds (mammalian) fucosides at the *N*-terminal domain and (bacterial) mannosides at the *C*-terminal domain. This double carbohydrate specificity allows the lectin to crosslink host cells and bacterial cells. We have recently reported the design and generation of the first glycomimetic antagonists of BC2L-C, β-*C*- or β-*N*-fucosides that target the fucose-specific *N-*terminal domain (BC2L-C-Nt). The low water solubility of the designed *N*-fucosides prevented a full examination of this promising series of ligands. In this work, we describe the synthesis and biophysical evaluation of new L-fucosyl and L-galactosyl amides, designed to be water soluble and to interact with BC2L-C-Nt. The protein–ligand interaction was investigated by Saturation Transfer Difference NMR, Isothermal Titration Calorimetry and crystallographic studies. STD-NMR experiments showed that both fucosyl and galactosyl amides compete with α-methyl fucoside for lectin binding. A new hit compound was identified with good water solubility and an affinity for BC2L-C-Nt of 159 μM (ITC), which represents a one order of magnitude gain over α-methyl fucoside. The x-ray structure of its complex with BC2L-C-Nt was solved at 1.55 Å resolution.

## 1. Introduction

Lectins are carbohydrate-binding proteins, widely distributed in Nature. They read out the signals associated with glycans and thus mediate numerous cellular recognition processes. In particular, microorganisms often use lectins to bind specific carbohydrates expressed on the outer membrane of host cells in a process that leads to cell adhesion [1]. Adhesion is a key step for pathogens to invade and infect host tissues, and thus has become the focus of much research directed at the improvement of anti-infective therapies. Indeed, bacterial infections are a major cause of mortality worldwide, and are becoming more and more threatening with the spread of anti-microbial resistance to antibiotics. Interference with adhesion (anti-adhesion therapy, AAT) is increasingly viewed as a viable way to prevent bacterial infections or to complement conventional antimicrobic treatments [2,3]. Many lectins have been identified as promising drug targets for AAT, and some of them have been validated, with clinical candidates in the pipeline [4,5,6].

**Figure 1:**
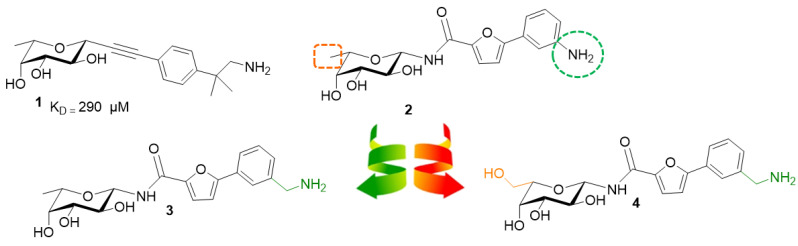
The BC2L-C-Nt ligand **1;** the water-insoluble amide **2** was found to occupy the same binding region as **1** in the X-ray of a complex obtained by ligand exchange in the crystal, but its affinity could not be measured [8]. Ligands **3** and **4** described in this paper are water-soluble analogs of **2**.

**Figure 2:**
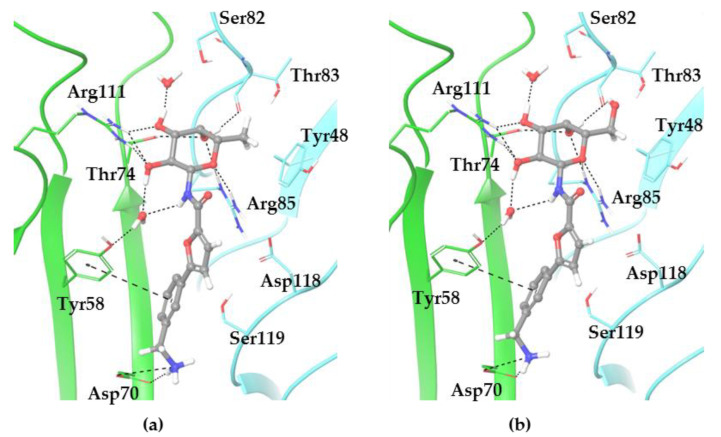
Expected binding mode of (**a**) **3** and (**b**) **4** in BC2L-C-Nt. The poses were obtained by docking using Glide (vers. 7.8), according to the protocol described in ref [8].

We have recently described the rational design and synthesis of a first group of antagonists of *Burkholderia cenocepacia* BC2L-C lectin, a candidate target for AAT against this pathogen, which is associated with fatal pulmonary infections of cystic fibrosis patients [7,8]. BC2L-C presents a double carbohydrate specificity, with a Man-specific carbohydrate recognition domain (CRD) in the *C*-terminal domain and a Fuc-specific CRD in the trimeric *N-*terminal domain [9,10]. Thus, BC2L-C can contribute to adhesion and colonization by simultaneously binding bacterial mannosides and human fucosides, and it has been dubbed a *superlectin* [9,10]. In our initial design, we targeted the BC2L-C *N*-terminal domain (BC2L-C-Nt) using bifunctional β-C- or β-N-fucosides (Figure 1, **1** and **2**). These ligands bear an aglycone moiety derived from fragments, which were predicted in silico to engage a small crevice close to the fucose-binding area. An evaluation of the new compounds, including BC2L-C-Nt ligands, resulted in a hit molecule, the C-glycoside **1** (Figure 1); this revealed an order of magnitude gain over the starting α-methyl fucoside, as judged by isothermal titration calorimetry [8]. X-ray crystallography strongly suggested that the fucosylamide **2** was also perfectly accommodated in the binding region and shared with **1** a number of stabilizing interactions with the protein surface. However, the affinity of **2** for BC2L-C-Nt could not be determined by ITC, owing to its very low water solubility. A direct interaction SPR assay, performed by immobilizing BC2L-C-Nt on a CM5 chip, yielded an apparent Kd 2.4 mM for **2**, but affinities measured in this assay did not correlate with ITC results for a series of other molecules examined, and could not be relied upon [8]. In order to verify the potential of the chemotype represented by ligand **2**, we set about to generate water soluble versions of **2** (Figure 1). In this paper, we report on their synthesis, interaction studies with BC2L-C-Nt by STD-NMR and affinity determination by ITC. Finally, the X-ray structure of the lectin complex of the most active of these new ligands is disclosed.

## 2. Results and Discussion

In order to increase the water solubility of **2**, while preserving its ability to interact with BC2L-C-Nt, we tackled two structural features, one in the carbohydrate moiety and one in the aglycone (Figure 1). On the aglycone side, we knew that the requirements for fitting the targeted crevice consist of a rather extended aromatic moiety, which generates a T-shaped interaction with Tyr58, and an amino group, targeting the side chain of Asp70 at the bottom end of the binding region [8]. We kept these features in the fucosylamide **3**, while transforming the aniline of **2** into an aminomethylene group. Indeed, it is well known that reducing the aromatic character of a molecule by increasing the number of saturated carbons tends to improve water solubility [11]. On the monosaccharide side, we capitalized on the observation that L-galactose binds to BC2L-C-Nt with an affinity that is similar to that of L-fucose [12] to draw up also the L-galactosylamide **4**. The docking of **3** and **4** in BC2L-C-Nt, using Glide (version 7.8) [13] with the established protocol [8], supported their ability to fit within the expected region and to generate extensive interactions with the protein (Figure 2). Thus, we set about to synthesize **3** and **4** and determine their activity as a BC2L-C ligand.

### 2.1. Synthesis

**Scheme 1:**
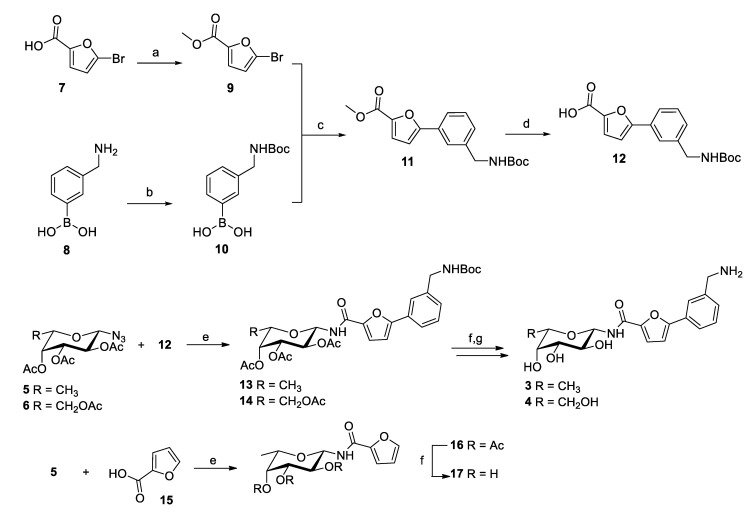
Synthesis of the ligands. Reagents and conditions: a. H_2_SO_4_ cat., MeOH, reflux, 18 h; b. Boc_2_O, TEA, THF, 0 → 25 °C, 10 h; c. PdCl_2_(PPh_3_)_2_, NaHCO_3_, DME/EtOH, 8–10 h; d. LiOH**^.^**H_2_O, THF/MeOH/H_2_O, rt, 2 h; e. PMe_3_, EDCI, HOAt, DIPEA, CH_2_Cl_2_, 0 → 25 °C, 18–20 h; f. MeONa, MeOH, rt, 2 h; g. TFA, CH_2_Cl_2_, rt, 1 h.

The synthetic pathway for the preparation of the target compounds involved the formation of two building blocks, the amino sugar core and the acid, which were subsequently joined by amide coupling (Scheme 1). Preparation of the carbohydrate moiety started from *β*-fucosyl azide (**5**) and *β*-galactosyl azide (**6**), which were synthesized by known strategies in the literature [14,15,16]. Full details about the synthesis of **5** and **6** are reported in Supplementary Materials (Section 1). The acid moiety was prepared starting from commercially available 5-bromo-2-furancarboxylic acid (**7**) and (3-(aminomethyl)phenyl)boronic acid (**8**, Scheme 1). The carboxylic acid was protected as a methyl ester (**9**), while the amino group in **8** was protected as Boc to afford **10**. Suzuki coupling of bromide **9** and boronic acid **10** (PdCl_2_(PPh_3_)_2_, NaHCO_3_) provided **11,** in good yield. Methyl ester hydrolysis (LiOH) afforded acid **12,** which was coupled to azide **5** or **6** using a Staudinger ligation (PMe_3_, EDCI and HOAt) to generate amides **13** or **14**. Zemplen deacetylation (MeONa) and TFA-induced cleavage of the Boc group gave the final compounds **3** and **4** (Scheme 1). Finally, as a control, the *N*-fucosyl-furanoylamide **17** was synthesized by Staudinger ligation of tetra-*O-*acetylfucosylazide **5** and commercially available furanoic acid **15**, followed by acetate hydrolysis on **16** (Scheme 1). To our delight, both **3** and **4**, as well as **17**, were fully soluble in water, at least up to 2 mM. 

### 2.2. Biophysical Evaluation

#### 2.2.1. STD NMR

Saturation Transfer Difference NMR (STD-NMR) experiments were used for an initial characterization of the interaction between BC2L-C-Nt and ligands **3** and **4**. 

STD-NMR is the most popular method to characterize events of molecular recognition for weakly bound ligands and it allows for the identification of the binding epitope, i.e., the parts of the ligand in closer contact with the protein. The method relies on the selective saturation of the protein by irradiation at an appropriate frequency (on-resonance frequency) and on the observation of the ligand signals, whose intensity will be modified by the transfer of magnetization from the protein protons. The degree of saturation of individual ligand protons, expressed as absolute or relative STD %, reflects their proximity to the protein surface (short protein–ligand distances produce a strong intensity of the corresponding STD signal) and can be used to describe the target–ligand interactions. The relative STD percentages can be calculated for each compound by normalizing all measured STD intensities against the most intense signal, which is arbitrarily assigned a value of 100%. The resultant epitope map can be influenced by the choice of saturation frequency [17].

**Figure 3:**
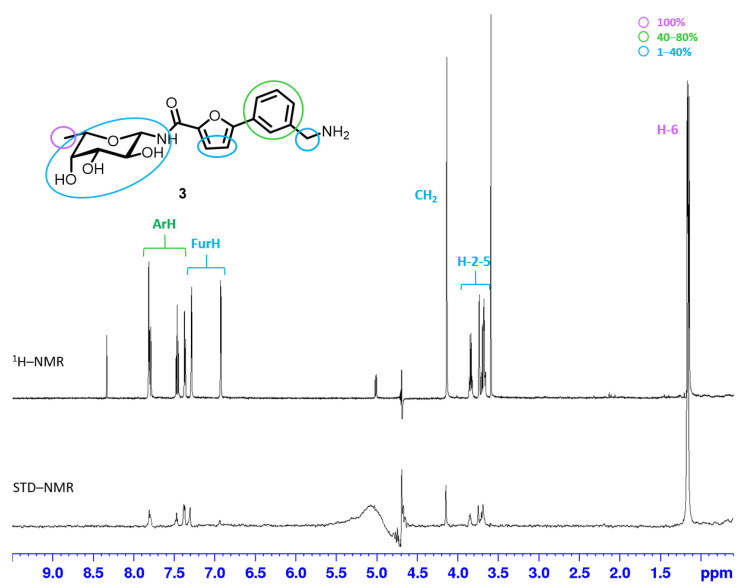
^1^H (top) and STD NMR (bottom) spectra for compound **3** in the presence of BC2L-C-Nt. The STD experiment was acquired on a Bruker Avance 600 MHz at 298 K, irradiating for 0.98 s at −0.05 ppm in phosphate buffer pH 7.4 with 10% D_2_O. The epitope is represented with a color code: purple circle for protons with 100% relative STD, green for 40–80% and light blue under 40%.

**Figure 4:**
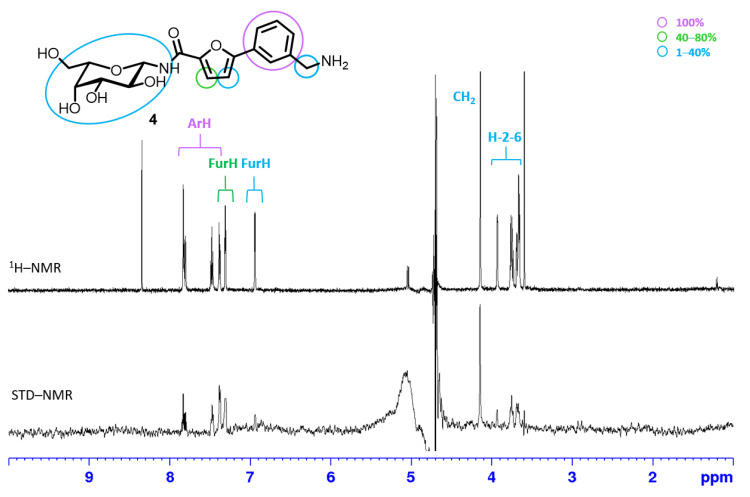
^1^H (top) and STD NMR (bottom) for compound **4** in presence of BC2L-C-Nt. The STD experiment was acquired on a Bruker Avance 600 MHz at 298 K, irradiating for 0.98 s at −0.05 ppm in phosphate buffer, pH 7.4 with 10% D_2_O. The epitope is represented with a color code: purple circle for protons with 100% relative STD, green for 40–80% and light blue under 40%.

STD spectra were obtained for each ligand in a pH 7.4 phosphate buffer in 9:1 H_2_O:D_2_O, by irradiating the protein at −0.05 ppm. A strong interaction was observed for both molecules. The spectra are reported in Figure 3 and Figure 4, and the calculated absolute and relative STD % are collected in Tables S1 and S2.

For compound **3**, the STD spectrum (Figure 3) revealed that the fucose ring is well inserted in the binding pocket and its C6-methyl group at 1.17 ppm gives rise to the strongest observable contact. The weaker response of the other protons of the sugar ring (3.5–4.0 ppm, rel. STD ca 20%) is consistent with the different nature of the interaction: the methyl group has a direct hydrophobic interaction with the protein residues, while for the rest of the ring, the contacts are mostly mediated by hydrogen bonds. The exchange between deuterons (from D_2_O) and the labile protons of both the ligand and protein is inevitable during the time scale of STD-NMR experiments, resulting in a lower STD intensity for protons involved in such interactions. A strong intensity was also observed for the protons of the aromatic ring (7.38–7.82 ppm, rel. STD 70%), while a moderate interaction could be inferred for the aminomethylene group (4.15 ppm, rel. STD 20%) and proton 3 of the furan ring (7.29 ppm, rel. STD 30%). 

The STD spectrum of compound **4** (Figure 4) suggests that it can bind BC2L-C with both sugar and aromatic moieties. In this case, the aromatic protons showed the strongest intensity, and a good interaction was also detected for proton 3 of the furan ring (7.31 ppm, rel. STD 63%) and for the aminomethylene group (4.14 ppm, rel. STD 31%). A weaker involvement was observed for the galactose moiety. 

A great deal of additional information can be gained from STD competition assays, where a known binder is added to a protein–ligand mixture with the aim of displacing the ligand under study and confirm that they are both competing for the same binding site. In the case at hand, α-methyl fucoside was used as a probe to validate the binding site of ligands **3** and **4,** confirming their ability to occupy the fucose-binding pocket of BC2L-C-Nt. α-methyl fucoside binds to BC2L-C with a Kd of 2.7 mM, as measured by ITC [9]. After the addition of α-methyl fucoside (1 mol equiv relative to **3**) to the BC2L-C-Nt:**3** complex, the STD signal of **3** decreased by 20–26% (Table S1), while a weak signal appeared in the STD spectrum for the α-methyl fucoside C-6 methyl group (at 1.12 ppm), showing that both ligands compete for the same binding site and that **3** has a stronger affinity than the methyl fucoside (Figure S1). The same competition experiment was performed for **4** in the presence of α-methyl fucoside. The reduction in STD intensity after the addition of the monosaccharide can be evaluated only for the aromatic protons, since the signals of galactose and methyl fucoside overlap in the region 3.8–3.6 ppm, and can be estimated around 60% (Table S2). This indicates that, also in this case, the two molecules occupy the same binding site and suggests that **4** is a stronger binder than α-methyl fucoside, but weaker than **3.**

#### 2.2.2. Isothermal Titration Calorimetry 

As compounds **3** and **4** were water soluble, their affinity for BC2L-C-Nt was determined by isothermal titration microcalorimetry (ITC) (Table 1). The affinities measured corroborate the NMR-STD results, showing that **3,** with a K_D_ of 159 µM, is only 3 times less potent than the H-type 1 tetrasaccharide, and is over one order of magnitude more potent than α-methyl fucoside. A comparison with the furanoyl amide **17** (K_D_ 2845 µM) clearly confirms the contribution of the aromatic moiety to the interaction that was visible in the STD spectra. The activity of the L-galactosyl derivative **4** (K_D_ 390 µM) is only lower by a factor of 2.5 than that of **3,** and this confirms that BC2L-C-Nt can accommodate a hydroxyl group on carbon 6 of the monosaccharide. All thermograms and fitted curves are depicted in Figures S2–S4.

### 2.3. X-ray Crystallography

In order to understand the atomic details of the interaction, the structure of BC2L-C-Nt was solved in complex with compound **3**. We soaked the co-crystals of BC2L-C-Nt in complex with H-type 1 or H-type 3, since the protein crystallized better in the presence of the ligand over 48 h. We collected data for both hexagonal and monoclinic crystal forms. Statistics on data and refinement are summarized in Table 2. The electron density revealed, without ambiguity, that ligand **3** displaced all of the blood group oligosaccharide. Only the hexagonal crystal form was refined at 1.55 Å, with one protomer in the asymmetric unit and the trimer forms, by applying the 3-fold crystallographic symmetry. The overall structure is equivalent to the one solved in complex with compound **2** (PDB ID 7OLW), with a core root mean square deviation of 0.1 Å over 131 amino acids. The main difference is at the *N*-terminus of the protein, where the first 3 residues (residual of the expression tag) were either too disordered to be built or may have been cleaved upon the aging of the crystals. As a result, the *C*-terminus is less stabilized and the electron density is of low quality, confirming disorder from Trp127. 

**Figure 5:**
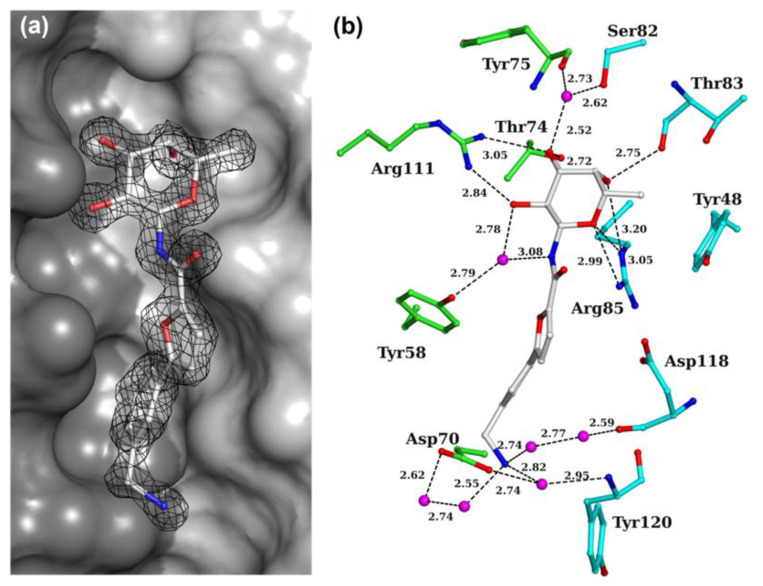
X-ray crystal structure of the BC2L-C-Nt:**3** complex at 1.55 Å. (**a**) Electronic density for ligand **3** in complex with BC2L-C-Nt represented at 1.3 σ (0.71 eÅ^3^). (**b**) Atomic detail of the interactions of **3** with BC2L-C-Nt (PDB ID 8BRO). Water molecules are depicted as purple spheres and direct or water-mediated hydrogen bonds are shown in black dashes. Protein carbon atoms are colored according to protomers.

All the interactions at the fucose moieties are equivalent to the one described in previous structures, with the binding site located in a shallow groove at the interface between protomers (Figure 5a). Binding interactions involve direct H-bonds of all the fucoside oxygens with Thr74, Arg85*, Thr83* and Arg111 (* amino acid from neighboring protomer). Two structural waters mediate H-bonds between O2 and O3 hydroxyls and Ser 82*, Tyr58 and Tyr75, and hydrophobic contacts are made with Tyr48* ring and the C6 methyl (Figure 5b). On the aglycone part, only the two nitrogen atoms are involved in hydrogen bonds mediated through water molecules. The glycosidic nitrogen is H-bonded to Tyr58 hydroxyl via one of the two structural waters described previously [7,8]. The terminal amine is interacting with the main chain oxygen of Asp118* via two water molecules, the main chain nitrogen of Tyr120* and OD1 atom of Asp70 via one water molecule, and with the OD2 atom of Asp70 via two water molecules (Figure 5b). All of these interactions block the position of the aminomethylene group.

**Figure 6:**
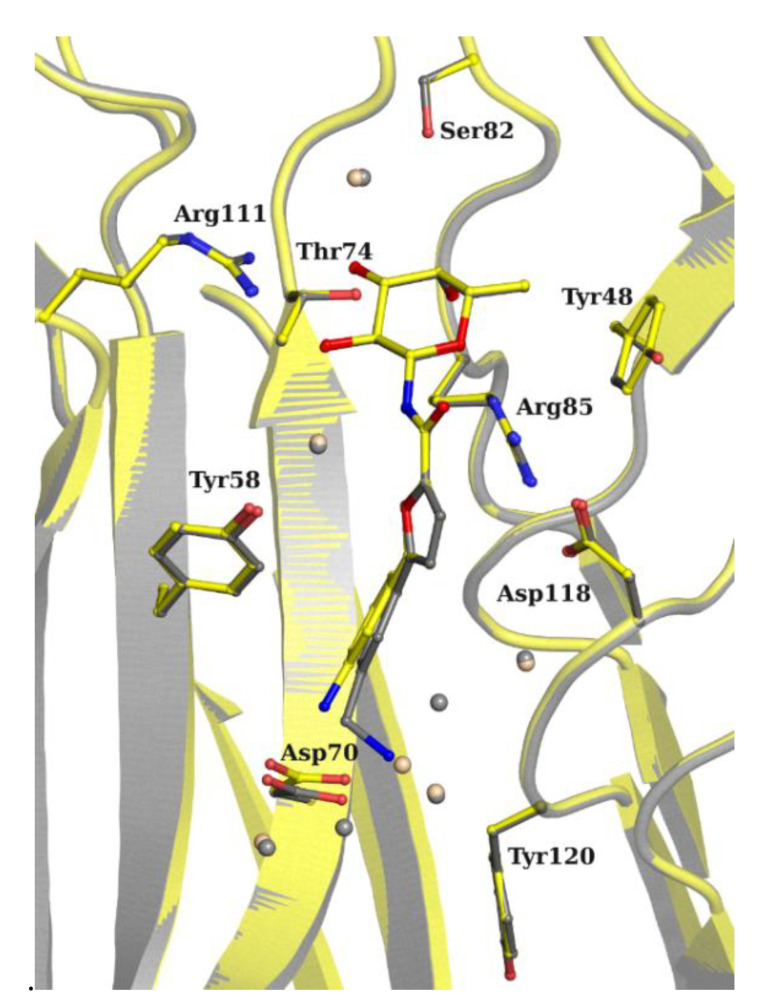
Overlay of the X-ray structure of the complexes of BC2L-C-Nt: with **2** (PDB 7OLW, yellow) and **3** (PDB 8BRO, grey).

The overlay of the structure of compounds **2** and **3** in their BC2L-C-Nt complex reveals only a shift at the level of the phenyl ring and of the side chain of Asp70 (Figure 6). The amine in compound **3** occupies the position of a water molecule in the complex of **2,** which was involved in mediating the interaction between the amine and the side chain of Asp70. The data are consistent with the docking predictions shown in Figure 2, although the absence of a water solvation model in the docking calculations tends to enforce a charge/charge interaction between the amino group of the ligand and the side chain of Asp70; this is dampened in the water environment for both **2** and **3**. A superimposition of the best docking pose with the X-ray structure is shown in Supplementary Materials (Figure S5). 

## 3. Materials and Methods

### 3.1. Synthesis

Reagents and anhydrous solvents were purchased from Sigma-Aldrich or abcr or Carbosynth. THF was dried over sodium/benzophenone and freshly distilled. Reactions were monitored by analytical thin-layer chromatography (TLC); this was performed on Silica Gel 60 F254 plates (Merck) and TLC Silica gel 60 RP-18 F254s (Merck), which were analyzed with UV detection (254 and 365 nm) and/or staining with ammonium molybdate acid solution, potassium permanganate alkaline solution, ninhydrin stain, and Dragendorff stain. Flash column chromatography was performed using silica gel 60 (40−63 μm, Merck). Automated flash chromatography was performed with a Biotage Isolera Prime system, and SNAP ULTRA cartridges were employed. For HPLC purifications, a Dionex Ultimate 3000, equipped with Dionex RS Variable Wavelength Detector (column: Atlantis Prep T3 OBDTM 5 µm 19 × 100 mm; flow 15 mL min−1 unless stated otherwise), was used at a flow rate of 10.0 mL/min. NMR experiments were carried out on a BrukerAVANCE 400 MHz instrument at 298 K. The ^13^C-NMR spectra are Attached Proton Test J-modulated spin-echo (APT). Mass spectra were recorded on a Thermo Fischer LCQ apparatus (ESI-MS). High resolution mass spectra were recorded on Synapt G2-S*i* QTof mass spectrometer (Waters)—Zspray ESI-probe (Waters) for electrospray ionization. Optical rotation values were measured using a Perkin-Elmer 241 polarimeter, at 589 nm in a 1dm cell. All compounds were purified by RP-HPLC before testing and their purity was ≥ 98%, as evaluated by 1H NMR. 

#### 3.1.1. Synthesis of Methyl 5-Bromofuran-2-Carboxylate (**9**)

To a solution of 5-bromo-2-furancarboxylic acid (250 mg, 1.31 mmol, 1 eq) in anhydrous methanol under N_2_ (4.5 mL, conc. = 0.3 M), conc. sulphuric acid (95–97%, 0.13 mL) was slowly added. The reaction mixture was stirred at reflux temperature until TLC showed the completion of the reaction (~18 h). The solvent was removed under reduced pressure. The residue was dissolved in dichloromethane (15 mL) and washed with water (7 mL), a sat. solution of NaHCO_3_ (7 mL) and brine. The organic layer was dried over anhydrous Na_2_SO_4_, filtered and concentrated to dryness, furnishing the desired product as a white solid. Y = 75%. Rf (*n*-Hex/AcOEt 1:1 + 0.1% formic acid) = 0.80. The characterization data are in agreement with those reported in the literature [18]. ^1^H NMR (400 MHz, CDCl_3_) δ 7.12 (d, *J* = 3.5 Hz, 1H, H-3), 6.45 (d, *J* = 3.5 Hz, 1H, H-4), 3.88 (s, 3H, -C*H_3_*).

#### 3.1.2. Synthesis of (3-(*tert*-Butoxycarbonyl)aminomethyl)phenylboronic Acid (**10**)

(3-(Aminomethyl)phenyl)boronic acid hydrochloride (150 mg, 0.80 mmol, 1 eq) was dissolved in anhydrous tetrahydrofuran (2.2 mL) and triethyl amine was added. The resulting mixture was stirred for 15 min, then it was cooled to 0 °C and a solution of di-*tert*-butyl dicarbonate (436 mg, 2 mmol, 2.5 eq) in anhydrous tetrahydrofuran (1 mL, final conc. = 0.25 M) was added. The reaction was stirred overnight at room temperature. The solvent was removed under vacuum and the residue was dissolved in ethyl acetate (5 mL) and washed with water (2 × 4 mL). The organic phase was dried over anhydrous Na_2_SO_4_, filtered and concentrated to dryness, obtaining the desired product as a white solid. Y = quant. Rf (CH_2_Cl_2_/MeOH 8:2) = 0.85. The characterization data are in agreement with those reported in the literature [19]. ^1^H NMR (400 MHz, CDCl_3_) δ 8.17–8.09 (m, 2H, H-2), 7.54 (d, *J* = 7.4 Hz, 1H, H-4), 7.48 (t, *J* = 7.4 Hz, 1H, H-5), 4.94 (brs, 1H, -NH), 4.45–4.40 (m, 2H, -CH_2_), 1.53 (s, 9H, *t*Bu).

#### 3.1.3. Synthesis of Methyl 5-(3-((*tert*-Butoxycarbonyl)aminomethyl)phenyl)furan-2-carboxylate (**11**)

To a solution of methyl 5-bromofuran-2-carboxylate **9** (115 mg, 0.56 mmol, 1 eq) in a mixture of anhydrous 1,2-dimethoxyethane (3.7 mL) and ethanol (3.7 mL) under N_2_ atmosphere, bis(triphenylphosphine)palladium(II) dichloride (20 mg, 0.028 mmol, 5%mol) was added. The reaction mixture was stirred for 30 min at room temperature, then an aqueous solution of NaHCO_3_ (282 mg, 3.36 mmol, 6 eq in 2.40 mL) was added, followed by a solution of (3-(*tert*-butoxycarbonyl)aminomethyl)phenylboronic acid **10** (156 mg, 0.62 mmol, 1.1 eq) in anhydrous ethanol (1.8 mL, final conc. = 0.06 M). The reaction was stirred at reflux temperature (85 °C) until TLC showed the full consumption of the starting materials (~10 h). Water (3 mL) was added to the mixture and extracted with ethyl acetate (2 × 10 mL). The organic layer was dried over anhydrous Na_2_SO_4_, filtered and concentrated under reduced pressure. The crude was purified by automatic chromatography (SFAR 10 g, *n*-Hex/AcOEt 8:2), obtaining the desired product as colorless oil. Y = 80%. Rf (*n*-Hex/AcOEt 3:1) = 0.41. MS (ESI) calcd for C_18_H_21_NO_5_ [M + Na]^+^ *m/z:* 354.13; found: 354.21. ^1^H NMR (400 MHz, CDCl_3_) δ 7.69–7.64 (m, 2H, Ph-H-2, Ph-H-6), 7.38 (t, *J* = 7.8 Hz, 1H, Ph-H-5), 7.30–7.21 (m, 2H, Ph-H-4, Fur-H-3), 6.74 (d, *J* = 3.6 Hz, 1H, Fur-H-4), 4.90 (brs, 1H, -N*H*), 4.38–4.33 (m, 2H, -C*H_2_*), 3.91 (s, 3H, COO*-*C*H_3_*), 1.47 (s, 9H, *t*Bu). ^13^C chemical shifts extrapolated from HSQC exp.: δ 129.2 (Ph-C-4), 128.3 (Ph-C-5), 124.1 (Ph-C-2, Ph-C-6), 120.4 (Fur-C-3), 107.1 (Fur-C-4), 51.7 (-O*C*H_3_), 28.4 (*t*Bu).

#### 3.1.4. Synthesis of 5-(3-((*Tert*-butoxycarbonyl)aminomethyl)phenyl)furan-2-carboxylic acid (**12**)

To a stirred solution of methyl 5-(3-((*tert*-butoxycarbonyl)aminomethyl)phenyl)furan-2-carboxylate **11** (110 mg, 0.33 mmol, 1 eq) in a ternary mixture of tetrahydrofuran/methanol/water (3:1:1, 1.3 mL), LiOH.H_2_O was added and the reaction mixture was stirred for 2 h at room temperature. The organic solvents were removed under reduced pressure. The aqueous phase was diluted with water (2 mL) and washed with ethyl acetate (4 mL), then it was acidified with 1M aqueous solution of HCl until pH 2. The acid aqueous layer was extracted with ethyl acetate (2 × 5 mL). The organic phase was washed with brine (4 mL), dried over anhydrous Na_2_SO_4_, filtered and concentrated under reduced pressure, to give the desired product as a white solid. Y = 77%. Rf (*n*-Hex/AcOEt 1:1 + 0.1% formic acid) = 0.46. MS (ESI) calcd for C_17_H_18_NO_5_ [M + Na]^+^ *m/z*: 340.12; found: 340.20. ^1^H NMR (400 MHz, CDCl_3_) δ 7.73–7.67 (m, 2H, Ph-H-2, Ph-H-6*),* 7.42–7.34 (m, 2H, Ph-H-5, Fur-H-3), 7.30 (d, *J* = 7.5 Hz, 1H, Ph-H-4), 6.78 (d, *J* = 3.6 Hz, 1H, Fur-H-4), 4.96 (brs, 1H, -N*H*), 4.39–4.32 (m, 2H, -C*H_2_*), 1.48 (s, 9H, *t*Bu). ^13^C chemical shifts extrapolated from HSQC exp.: 128.6 (Ph-C-5, Ph-C-4), 123.8 (Ph-C-2, Ph-C-6), 121.3 (Fur-C-3), 106.8 (Fur-C-4), 44.2 (-*C*H_2_), 27.8 (*t*Bu). 

#### 3.1.5. General Procedure for the Synthesis of Compounds **13**, **14** and **16** through Staudinger Ligation

5-(3-((*tert*-butoxycarbonyl)aminomethyl)phenyl)furan-2-carboxylic acid **12** (54 mg, 0.17 mmol, 1 eq) was suspended in anhydrous dichloromethane (1.7 mL, conc. = 0.1 M) and 1-ethyl-3-(3-dimethylaminopropyl)carbodiimide (36 mg, 0.19 mmol, 1.1 eq) was added, together with 1-hydroxy-7-azabenzotriazole (25 mg, 0.19 mmol, 1.1 eq) and *N,N*-diisopropylethylamine (0.074 mL, 0.43 mmol, 2.5 eq). The resulting mixture was stirred at room temperature for 1 h under N_2_. (2,3,4-tri-*O*-acetyl β-L-fucopyranosyl) azide **5** (76 mg, 0.24 mmol, 1.4 eq) or 2,3,4,6-tetra-*O*-acetyl-β-L-galactopyranosyl azide **6** were dissolved in anhydrous dichloromethane (1.6 mL, conc. = 0.15 M) and cooled to 0 °C under N_2_. Trimethylphosphine (0.27 mL, 0.27 mmol, 1.6 eq) was slowly added and the reaction mixture was stirred at room temperature for 20 min. The activated acid mixture was combined to the reduced azide one and the whole reaction was stirred overnight at room temperature. It was diluted with dichloromethane (2 mL) and washed with sat. solution of NH_4_Cl (2 × 3 mL), sat. solution of NaHCO_3_ (3 mL) and brine (3 mL). The organic layer was dried over anhydrous Na_2_SO_4_, filtered and concentrated to dryness. The product was purified by automatic chromatography, using *n*-Hex/AcOEt as eluent. 

##### (5-(3-((*Tert*-butoxycarbonyl)aminomethyl)phenyl)furan-2-carboxamido)-2,3,4-tri-O-acetyl-β-L-fucopyranose (**13**)

The product was purified by automatic chromatography (SFAR 5 g, *n*-Hex/AcOEt 6:4) to give the desired product as yellow oil. Y = 53%. Rf (CH_2_Cl_2_/MeOH 98:2) = 0.41. MS (ESI) calcd for C_29_H_36_N_2_O_11_ [M + Na]^+^ *m/z*: 611.22; found: 611.39.^1^H NMR (400 MHz, CDCl_3_) δ 7.64 (s, 1H, Ph-H-2), 7.59 (d, *J* = 7.7 Hz, 1H, Ph-H-6), 7.38–7.26 (m, 2H, Ph-H-5, -N*H*), 7.23 (d, *J* = 7.5 Hz, 1H, Ph-H-4), 7.16 (d, *J* = 3.5 Hz, 1H, Fur-H-3), 6.67 (d, *J* = 3.5 Hz, 1H, Fur-H-4), 5.33–5.18 (m, 3H, H-1, H-2, H-4), 5.14 (dd, *J* = 9.7, 3.1 Hz, 1H, H-3), 4.98 (s, 1H, -N*Ht*Bu), 4.32 (m, 2H, -C*H_2_*), 3.96 (q, *J* = 6.2 Hz, 1H, H-5), 2.14 (s, 3H, OAc), 1.98 (s, 3H, OAc), 1.95 (s, 3H, OAc), 1.40 (s, 9H, *t*Bu), 1.16 (d, *J* = 6.4 Hz, 3H, H-6). ^13^C chemical shifts extrapolated from HSQC exp.: 129.4 (Ph-C-5), 128.2 (Ph-C-4), 123.7 (Ph-C-2, Ph-C-6), 117.6 (Fur-C-3), 107.4 (Fur-C-4), 78.7 (C-1), 70.6 (C-3, C-2, C-5), 68.5 (C-4), 28.7 (*t*Bu), 20.8 (OAc), 16.3 (C-6). 

##### (5-(3-((*Tert*-butoxycarbonyl)aminomethyl)phenyl)furan-2-carboxamido)-2,3,4,6-tetra-O-acetyl-β-L-galactopyranose (**14**)

The product was purified by automatic chromatography (SFAR 5 g, *n*-Hex/AcOEt 5:5 to 4:6) to give the desired product as yellow oil. Y = 40%. Rf (CH_2_Cl_2_/MeOH 98:2) = 0.65. MS (ESI) calcd for C_31_H_38_N_2_O_13_ [M + Na]^+^ *m/z*: 669.23; found: 611.40.^1^H NMR (400 MHz, CDCl_3_) δ 7.71 (s, 1H, Ph-H-2), 7.65 (d, *J* = 7.6 Hz, 1H, Ph-H-6), 7.45–7.32 (m, 2H, Ph-H-5, -N*H*), 7.29 (d, *J* = 7.5 Hz, 1H, –Ph-H-4), 7.23 (d, *J* = 3.6 Hz, 1H, Fur-H-3), 6.74 (d, *J* = 3.6 Hz, 1H, Fur-H-4), 5.49 (d, *J* = 3.1 Hz, 1H-4), 5.48–5.32 (m, 1H, H-1), 5.30–5.27 (m, 1H, H-2), 5.21 (dd, *J* = 9.9, 3.3 Hz, 1H, H-3), 5.04 (brs, 1H, -N*Ht*Bu), 4.40–4.36 (m, 2H, -C*H_2_*), 4.18–4.07 (m, 3H, H-6, H-6′, H-5), 2.17 (s, 3H, OAc), 2.08–1.97 (m, 9H, OAc), 1.46 (d, *J* = 17.8 Hz, 9H, *t*Bu). ^13^C chemical shifts extrapolated from HSQC exp.: 129.5 (Ph-C-5), 128.3 (Ph-C-4), 123.8 (Ph-C-2, Ph-C-6), 117.7 (Fur-C-3), 107.7 (Fur-C-4), 78.7(C-1), 72.6 (C-5), 70.8 (C-3), 68.3 (C-2), 67.2 (C-4), 28.4 (*t*Bu), 20.9 (OAc).

##### (Furan-2-carboxamido)-2,3,4-tri-O-acetyl-β-L-fucopyranose (**16**)

The product was purified by automatic chromatography (SFAR 10 g, *n*-Hex/AcOEt 8:2→6:4) to give the desired product as yellow oil. Y = 95%. Rf (*n*-Hex/AcOEt 4:6) = 0.43. Characterization data in agreement with those reported in literature [20]. MS (ESI) calcd for C_17_H_21_NO_9_ [M + Na]^+^ *m/z*: 406.11; found: 406.66. ^1^H NMR (400 MHz, CDCl_3_) ^1^H NMR (400 MHz, CDCl_3_) δ 7.42 (d, *J* = 0.8 Hz, 1H, Ar-H-5), 7.10–7.05 (m, 2H, Ar-H-3, -N*H*), 6.44 (dd, *J* = 3.4, 1.7 Hz, Ar-H-4), 5.32–5.22 (m, 2H, H-1, H-4), 5.21–5.07 (m, 2H, H-2, H-3), 3.93 (q, *J* = 6.4 Hz, 1H, H-5), 2.12 (s, 3H, OAc), 1.95 (d, *J* = 6.2 Hz, 6H, OAc), 1.14 (d, *J* = 6.4 Hz, 3H, H-6). ^13^C chemical shifts extrapolated from HSQC exp.: δ = 144.9 (Ar-C-5), 115.8 (Ar-C-3), 112.2 (Ar-C-4), 78.2 (C-1), 71.4 (C-3), 70.4 (H-4, H-5), 68.1 (H-2), 20.6 (OAc), 16.2 (C-6). 

#### 3.1.6. General Procedure for Synthesis of Compounds **3** and **4** and **17**

##### General Procedure for Zemplén Deacetylation

Here, **13** or **14** or **16** (0.11 mmol, 1 eq) were dissolved in anhydrous methanol (0.55 mL, conc. = 0.2 M) under N_2_, and a 0.1 M solution of sodium methoxide in anhydrous methanol (0.05 mL) was added (final conc. of methoxide = 0.01 M). The resulting mixture was stirred at room temperature until full conversion (~2 h). The reaction was quenched by adding Amberlite IRA 120 H^+^ until pH 7, then the resin was filtered out and the solvent was removed under vacuum to give the desired product; this was used without further purification for Boc removal.

##### General Procedure for Boc Removal

The deacetylation crude (0.11 mmol, 1 eq) was dissolved in anhydrous dichloromethane (11 mL) under N_2_, and trifluoroacetic acid (1.2 mL) was added. The reaction mixture was stirred at room temperature until TLC showed the full conversion (1 h). The solvent was removed under reduced pressure and co-evaporated with methanol. The final compound was purified by HPLC using acetonitrile/water (+ 0.1% formic acid) as eluent. 

##### Synthesis of (5-(3′-Aminomethyl)phenyl)furan-2-carboxamido)-β-L-fucopyranose (**3**)

**13** was subjected to standard deacetylation and the crude was submitted to the following step. 

An analytical sample was used for the characterization of the intermediate *(5-(3-((tert-butoxycarbonyl)aminomethyl)phenyl)furan-2-carboxamido)-β-L-fucopyranose:* Colourless oil. Y = 98%. Rf (CH_2_Cl_2_/MeOH 93:7) = 0.22. MS (ESI) calcd for C_23_H_30_N_2_O_8_ [M + Na]^+^ *m/z*: 485.19; found: 485.40.^1^H NMR (400 MHz, MeOD) δ 7.80–7.75 (m, 2H, Ph-H-2, Ph-H-6), 7.41 (t, *J* = 7.6 Hz, 1H, Ph-H-5), 7.33–7.25 (m, 2H, Ph-H-4, Fur-H-3), 6.93 (d, *J* = 3.6 Hz, 1H, Fur-H-4), 5.08 (d, *J* = 9.0 Hz, 1H, H-1), 4.29 (s, 2H, -C*H_2_*), 3.82–3.72 (m, 2H, H-5, H-2), 3.69 (d, *J* = 2.9 Hz, 1H, H-4), 3.59 (dd, *J* = 9.5, 3.3 Hz, 1H, H-3), 1.47 (s, 9H, *t*Bu), 1.27 (d, *J* = 6.5 Hz, 3H, H-6). ^13^C chemical shifts extrapolated from HSQC exp.: 129.1 (Ph-C-5), 127.4 (Ph-C-4), 123.4 (Ph-C-2, Ph-C-6), 117.2 (Fur-C-3), 107.1 (Fur-C-4), 80.4 (C-1), 74.9 (C-3), 72.0 (C-4, C-5), 69.8 (C-2), 27.6 (*t*Bu), 15.7 (C-6).

Boc deprotection of the deacetylation crude afforded **3** as a white foam. Y = 95%. Rf (CH_2_Cl_2_/MeOH 8:2) = 0.24. The product was further purified by semi-preparative RP-HPLC (H_2_O + 0.1% formic acid/CH_3_CN) with gradient: 0–2 min: 0%, 2–17 min: 0–20%. t_r_ = 13.06 min. [α]_D_^18^ = 5.5 (c 1, MeOH). MS (ESI) calcd for C_18_H_22_N_2_O_6_ [M + H]^+^ *m/z*: 363.16; found: 363.27. HRMS (ESI) calculated for C_18_H_22_N_2_O_6_ [M + Na]^+.^ m/z: 385.1376; found 385.1376.^1^H NMR (400 MHz, MeOD) δ 7.95 (s, 1H, Ph-H-2), 7.90 (d, *J* = 7.7 Hz, 1H, Ph-H-6), 7.51 (t, *J* = 7.7 Hz, 1H, Ph-H-5), 7.44 (d, *J* = 7.7 Hz, 1H, Ph-H-4), 7.28 (d, *J* = 3.6 Hz, 1H, Fur-H-3), 6.96 (d, *J* = 3.6 Hz, 1H, Fur-H-4), 5.09 (d, *J* = 9.0 Hz, 1H, H-1), 4.18 (s, 2H, -C*H_2_*), 3.83–3.75 (m, 2H, H-5, H-2), 3.70 (d, *J* = 3.1 Hz, 1H, H-4), 3.61 (dd, *J* = 9.5, 3.3 Hz, 1H, H-3), 1.26 (d, *J* = 6.4 Hz, 3H, H-6). ^13^C NMR (100 MHz, MeOD) δ 159.7 (-*C*ONH), 155.6 (Fur quat), 146.4 (Fur quat), 133.9 (Ar quat), 130.4 (Ar quat), 129.8 (Ph-C-5), 128.8 (Ph-C-4), 124.9 (Ph-C-2), 124.8 (Ph-C-6), 117.0 (Fur-C-3), 107.6 (Fur-C-4), 80.1 (C-1), 74.7 (C-3), 72.8 (C-2), 72.0 (C-4), 69. (C-5), 42.8 (-*C*H_2_), 15.6 (C-6). [α]_D_^17.6^ = 5.5 (MeOH)

##### Synthesis of (5-(3′-Aminomethyl)phenyl)furan-2-carboxamido)-β-L-galactopyranose (**4**)

**14** was subjected to standard deacetylation and the crude was submitted to the following step. 

An analytical sample was used for the characterization of the intermediate *(5-(3-((tert-butoxycarbonyl)aminomethyl)phenyl)furan-2-carboxamido)-β-L-galactopyranose:* Colourless oil. Y = 85%. Rf (CH_2_Cl_2_/MeOH 93:7) = 0.22. MS (ESI) calcd for C_23_H_30_N_2_O_9_ [M + Na]^+^ *m/z*: 501.18; found: 501.31.^1^H NMR (400 MHz, CDCl_3_) δ 7.79–7.74 (m, 2H, Ph-H-2, Ph-H-6), 7.40 (t, *J* = 7.6 Hz, 1H, Ph-H-5), 7.30–7.27 (m 2H, Ph-H-4, Fur-H-3), 6.92 (d, *J* = 3.6 Hz, 1H, Fur-H-4), 5.10 (d, *J* = 9.0 Hz, 1H, H-1), 4.27 (s, 2H, -C*H_2_*), 3.93 (d, *J* = 3.0 Hz, 1H, H-4), 3.84–3.63 (m, 4H, H-2, H-5, H-6, H-6′), 3.59 (dd, *J* = 9.5, 3.3 Hz, 1H, H-3), 1.45 (s, 9H, *t*Bu). ^13^C chemical shifts extrapolated from HSQC exp.: 132.8 (Ph-C-5), 131.5 (Ph-C-4), 127.4 (Ph-C-2, Ph-C-6), 121.2 (Fur-C-3), 110.9 (Fur-C-4), 84.4 (C-1), 81.2 (C-2), 78.5 (C-3), 73.3 (C-4, C-5), 31.7 (*t*Bu).

Boc deprotection of the deacetylation crude afforded **4** as a white foam. Y = quant.. Rf (CH_2_Cl_2_/MeOH 7:3) = 0.20. The product was further purified by semi-preparative RP-HPLC (H_2_O + 0.1% FA/CH_3_CN) with gradient: 0–2 min: 0%, 2–12 min: 0–10%. t_r_ = 12.12 min. [α]_D_^18^ = 7.3 (c 1, MeOH). MS (ESI) calcd for C_18_H_22_N_2_O_7_ [M + H]^+^ *m/z*: 379.15; found: 379.50. HRMS (ESI) calculated for C_18_H_22_N_2_O_7_ [M + H]^+.^ m/z: 379.1505; found 379.1507. ^1^H NMR (400 MHz, MeOD) δ 8.56 (s, 1H, FA), 8.01 (s, 1H, Ph-H-2), 7.95 (d, *J* = 7.8 Hz, 1H, Ph-H-6), 7.55 (t, *J* = 7.7 Hz, 1H, Ph-H-5), 7.47 (d, *J* = 7.5 Hz, 1H, Ph-H-4), 7.34 (d, *J* = 3.6 Hz, 1H, Fur-H-3), 7.02 (d, *J* = 3.6 Hz, 1H, Fur-H-4), 5.14 (d, *J* = 9.0 Hz, 1H, H-1), 4.20 (s, 2H, -C*H_2_*), 3.97 (d, *J* = 2.9 Hz, 1H, H-4), 3.85–3.68 (m, 4H, H-2, H-5, H-6, H-6′), 3.62 (dd, *J* = 9.5, 3.2 Hz, 1H, H-3). ^13^C NMR (100 MHz, MeOD) δ 159.8 (-*C*ONH), 155.6 (Fur quat), 146.4 (Fur quat), 133.9 (Ar quat), 130.4 (Ar quat), 129.5 (Ph-C-5), 129.0 (Ph-C-4), 124.9 (Ph-C-2), 124.8 (Ph-C-6), 117.0 (Fur-C-3), 107.6 (Fur-C-4), 80.3 (C-1), 77.1 (C-2), 74.6 (C-3), 69.8 (C-5), 69.4 (C-4), 61.3 (C-6, C-6′), 42.8 (-*C*H_2_). 

##### Synthesis of Furan-2-carboxamido-β-L-fucopyranose (**17**)

**16** was subjected to standard deacetylation to afford **17** as a white foam. Y = 90%. Rf (nHex:EtOAc=95:5) 0.1. Characterization data in agreement with those reported in the literature [20]. MS (ESI) calcd for C_11_H_15_NO_6_ [M + Na]^+^ *m/z*: 280.08; found: 280.35. ^1^H NMR (400 MHz, MeOD) δ 7.59 (d, *J* = 0.9 Hz, 1H, Ar-H-5), 7.11 (d, *J* = 3.4 Hz, 1H, Ar-H-3), 6.50 (dd, *J* = 3.5, 1.7 Hz, 1H, Ar-H-4), 4.94 (d, *J* = 9.0 Hz, 1H, H-1), 3.67 (q, *J* = 6.4 Hz, 1H, H-5), 3.63–3.56 (m, 2H, H-4, H-2), 3.49 (dd, *J* = 9.5, 3.3 Hz, 1H, H-3), 1.16 (d, *J* = 6.5 Hz, 3H, H-6). ^13^C chemical shifts extrapolated from HSQC exp.: δ = 145.2 (C-5), 114.7 (C-3), 111.5 (C-4), 80.0 (C-1), 74.5 (C-3), 72.6 (C-5), 71.8 (C-4), 69.6 (C-2), 15.4 (C-6). 

### 3.2. STD NMR

NMR experiments were performed on 400 MHz Bruker Avance III and 600 MHz Bruker Avance spectrometers at 298 K in deuterated or aqueous phosphate buffer at pH 7.4. When required, water suppression was achieved by excitation sculpting sequence from standard Bruker library. STD NMR experiments were performed using WATERGATE 3-9-19 pulse sequence for water suppression. On-resonance irradiation of the protein was performed at a chemical shift of −0.05 ppm; off-resonance irradiation was applied at 200 ppm, where no protein signals are visible. Selective pre-saturation of the protein was achieved by a train of Gauss-shaped pulses of 49 ms in length each. The STD spectra were acquired with a ligand:protein ratio 1000:1, using 0.98 s and 2.94 s as saturation time. Blank experiments were acquired in absence of protein in order to avoid artefacts. Intensities of all STD effects (absolute STD) were calculated by division through integrals over the respective signals in STD reference spectra. The different signal intensities of the individual protons are best analyzed from the integral values in the reference and STD spectra, respectively.

$\frac{I_{0-I_{\mathrm{sat}}}}{I_{0}}\;$ is the fractional STD effect, expressing the signal intensity in the STD spectrum as a fraction of the intensity of an unsaturated reference spectrum. In this equation, I_0_ is the intensity of one signal in the off-resonance or reference NMR spectrum, I_sat_ is the intensity of a signal in the on-resonance NMR spectrum, and I_0_ − I_sat_ represents the intensity of the STD spectrum.

In order to map the epitope of the analyzed molecules to BC2L-C-Nt, the relative STD percentages were also calculated by comparing the STD intensities to the strongest signal, which was given an arbitrary value of 100%. The relative STD percentages are shown on the group epitope map using the following color code: purple circles are used to highlight the strongest signal (100% relative STD), green circles are for protons between 40 and 80%, and light blue circles are for signals under 40% relative to the strongest STD signal. 

### 3.3. Isothermal Microcalorimetry

All measurements were performed at 25 °C using an ITC200 isothermal titration calorimeter (MicroCal-Malvern Panalytical, Palaiseau, France). The protein and the different ligands were dissolved in a buffer composed of 20 mM Tris HCl pH 7.0 and 100 mM NaCl. The 200 μL sample cell containing the protein at a concentration ranging from 119 to 127 μM was subjected to 39 injections of 1 μL of 30 mM ligand solution (**3** and **4**), with intervals of 100 s while stirring at 850 rpm. For compound **17**, the protein concentration was between 139 and 187 µM, and the ligand concentration was 50 mM. The supplied software MicroCal PEAQ-ITC (Malvern Panalytical, Palaiseau, France) was used to fit the experimental data to a theoretical titration curve using one set of site models; the stoichiometry was fixed to 1 as all measurements were performed in an excess of ligand. We were able to determine the affinity (i.e., association constant, Ka) and the binding enthalpy (ΔH). The quality of the protein batches was controlled by titrating with 3mM H-type 1 tetrasaccharide (Fucα1-2Galβ1-3GlcNAcβ1-3Gal). A K_D_ of 56 ± 4.5 µM was obtained and was equivalent to that described previously. We estimated from the stoichiometry that 80% of the protein was actively binding and, therefore, protein concentration was corrected accordingly. (see Figures S2–S4). Control experiments were performed by repeating the same protocol, but injecting the ligand into the buffer solution to take into account buffer mismatch; this signal was subtracted in the fitting step. 

### 3.4. X-ray Crystallography

Cocrystals of BC2L-C-Nt in complex with H-type 1 trisaccharide were obtained as described previously [11]. Then, 18-month-old crystals were transferred and soaked in the cryoprotecting solution containing 2.47 M sodium malonate, pH 7.0, and 3 mM of compound **3** for 48 h at 19 °C. Single crystals were then directly mounted in a cryoloop (Molecular Dimensions, Calibre Scientific UK, Rotherham) and flash cooled in liquid nitrogen. Data were collected on Proxima 1 beamline at the synchrotron SOLEIL, Saint Aubin, France using an Eiger 16M detector (see statistics in Table 2). XDS and XDSME were used to process the data and all further steps were performed using programs of the CCP4 suite version 8.25–27 [21,22,23]. The structure of BC2L-C-Nt in complex with compound **3** was solved by molecular replacement using the protein coordinates of PDB-ID 6TID as search model in PHASER [24]. Iterated restrained maximum likelihood refinement and manual building were performed using REFMAC 5.8 and Coot, respectively. Hydrogen atoms were added in their riding positions during refinement and cross validation was performed with 5% of the observations set aside [25,26]. The library for the ligand VJW was used as a model in Jligand to create the library for compound **3**. The coordinates were deposited in the Protein Data Bank (PDB) under code 8BRO.

## 4. Conclusions

The results discussed above represent the first quantitative evaluation of the β-fucosylamide chemotype as a ligand of the *B. cenocepacia* lectin BC2L-C-Nt. In order to obtain these data, we had to overcome the low water solubility of initial hits, such as **2**, that were found to interact in the targeted binding site by X-ray crystallography, but whose affinity could not be properly assessed. This is a common issue for glycomimetics, which are often designed by exploiting the lipophilic interaction potential of aromatic substructures, together with the lectin-targeting ability of specific monosaccharides. Thus, the approach we followed here can be of general interest for the design of viable glycomimetic ligands. Water solubility was pursued by a) reducing the “flatness” of the structure by turning an aniline into a benzylamine moiety, and b) replacing L-fucose with L-galactose. While the latter move reduced the affinity of the ligand by a factor of 2.5, as measured by ITC, it still produced a ligand, compound **4**, which stands among the most active glycomimetics described so far against BC2L-C-Nt. STD-NMR data showed that **4** competes for binding with α-methyl fucoside, and thus suggests that the lectin can tolerate a hydroxyl group in the binding region used by fucose methyl group. This is a useful indication because, beside increasing solubility, the additional hydroxyl group of L-galactose can provide a handle for the conjugation of the ligand, for instance, to polyvalent scaffolds. The L-fucosylamide **3**, with a K_D_ 159 µM, is one of the most potent glycomimetic ligands of BC2L-C-Nt described so far, and presents a great potential for structure activity relationship (SAR) studies, given the optimal synthetic accessibility of the chemotype. All the available data indicate that, as previously observed in the β-fucosylalkyne series, represented by **1**, lipophilic interactions of the aromatic fragment within the targeted crevice at the monomer interface of the lectin are paramount in establishing the ligand affinity. The terminal amino group helps by interacting with the side chain of Asp70, at the opposite end of the crevice from the sugar. This interaction occurs near the surface of the protein and, as shown by the available X-ray structures, it cannot be described as the charge/charge interaction predicted by docking calculations, but rather as a water mediated contact, that can extend to other elements of the protein surface. All together, these studies are amassing a series of information and SAR that will be instrumental for the design of high affinity ligands to be tested in the disruption of *B. cenocepacia* biofilm.

## Figures and Tables

**Table 1:** Affinity of carbohydrate ligands of BC2L-C-Nt ^a,b^.

Entry	Ligand		n	K_D_ (μM)
1	H-type 1 tetrasaccharide	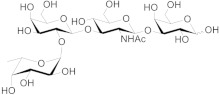	0.81	56 ± 4.5
2	**3**	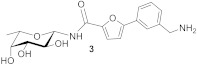	1	159 ± 7
3	**4**	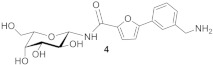	1	390.5 ± 15.5
4	**17**	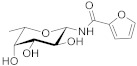	1	2845 ± 45
5	α-methyl fucoside	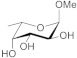		2700 ± 700 ^c^

^a^ Experiments performed at 25 °C. Averages and experimental errors for at least two independent measurements. ^b^ Stoichiometry was fixed to 1, and the blank of the titration of the ligand in buffer was subtracted during the fitting procedure: thermodynamic values cannot be assessed. ^c^ from ref. [9].

**Table 2:** Data collection and refinement statistics. Values in brackets are for highest-resolution shell.

Data Collection		Refinement	
Beamline	SOLEIL Proxima-1	Resolution (Å)	37.25–1.55
Wavelength	0.97856	No. reflections	13,457
Space group	P6_3_	No. free reflections	734
Unit cell dimensions	43.01 43.01 94.22 90.00 90.00 120.00	*R*_work_/*R*_free_	0.142/0.169
Resolution (Å)	37.25–1.55 (1.58–1.55)	R.m.s Bond lengths (Å)	0.0153
*R*_merge_ **	0.064 (0.447)	Rmsd Bond angles (°)	1.821
R_meas_ **	0.076 (0.540)	Rmsd Chiral (Å^3^)	0.103
*R_pim_* **	0.040 (0.297)	Rmsd Chiral (Å^3^)	0.103
*Mean I*/σ*I*	17.8 (3.5)	Clashscore	2.49
Completeness (%)	99.1 (99.7)	No. atoms/Bfac (Å^2^)	
Redundancy	5.6 (4.6)	Protein	993/11.53
CC1/2	0.998 (0.888)	Ligand	26/8.97
Nb reflections	79,558 (3313)	Water	128/21.57
Nb unique reflections	14,192 (716)	Ramachandran (%)	
		Allowed	100
		Favored	97.1
		Outliers	0
		PDB Code	8BRO

** value (within I^+^/I^−^).

## Data Availability

The crystallographic data are publicly available from the PDB website using the 8BRO ID code.

## Supplementary Materials

The following supporting information can be downloaded at: https://www.mdpi.com/article/10.3390/molecules28031494/s1, Figure S1. Competition experiment between **3** and α-methyl fucoside; Figure S2: Isothermal titration calorimetry titration of BC2L-C-Nt by **3**; Figure S3: Isothermal titration calorimetry titration of BC2L-C-Nt by **4**; Figure S4: Isothermal titration calorimetry titration of BC2L-C-Nt by **17**; Figure S5: Comparison between X-ray and docked structure of **3**; Table S1. Absolute and relative STD % for compound **3**; Table S2. Absolute and relative STD % for compound **4**; ^1^H and ^13^C-NMR or HSQC of all new compounds.
