# Supplementary material for: Identification of New L-Fucosyl and L-Galactosyl Amides as Glycomimetic Ligands of TNF Lectin Domain of BC2L-C from Burkholderia cenocepacia
Source: Molecules. 2023 Feb 3;28(3):1494. doi: 10.3390/molecules28031494 (PMC9919437; doi:10.3390/molecules28031494)
Supplement: Supplementary file 1 [file molecules-28-01494-s001.zip › molecules-2132450-supplementary.pdf]

Supplementary Materials

Identification of new L-fucosyl and L-galactosyl amides as glycomimetic ligands of TNF lectin domain of BC2L-C from *Burkholderia cenocepacia*

Sarah Mazzotta[1], Giulia Antonini[1], Francesca Vasile[1], Emilie Gillon[2], Jon Lundstrøm[2,3], Annabelle Varrot[2], Laura Belvisi[1], Anna Bernardi[1]

1 Università degli Studi di Milano, Dipartimento di Chimica; Milano, Italy
2 Univ. Grenoble Alpes, CNRS, CERMAV, 38000 Grenoble, France;
3 present address: Department of Chemistry and Molecular Biology, University of Gothenburg, Gothenburg, Sweden / Wallenberg Centre for Molecular and Translational Medicine, University of Gothenburg, Gothenburg, Sweden

Contents

1. Synthetic procedures and chemical characterization S2
1.1. Synthesis of *β*-L-fucosyl azide (**5**) S2
1.2. Synthesis of β-L-galactosyl azide (**6**) S3
2. STD NMR S4
2.1 Competition experiments S4
Figure S1. S4
2.2 Absolute and relative STD % for compounds **3** and **4** S5
Table S1. S5
Table S2. S6
3. Isothermal titration calorimetry S7
Figure S2. S7
Figure S3. S7
Figure S4. S8
4. Comparison of X-ray and docked structure S9
5. NMR spectra of new compounds S10
References S18

1. Synthetic procedures and chemical characterization

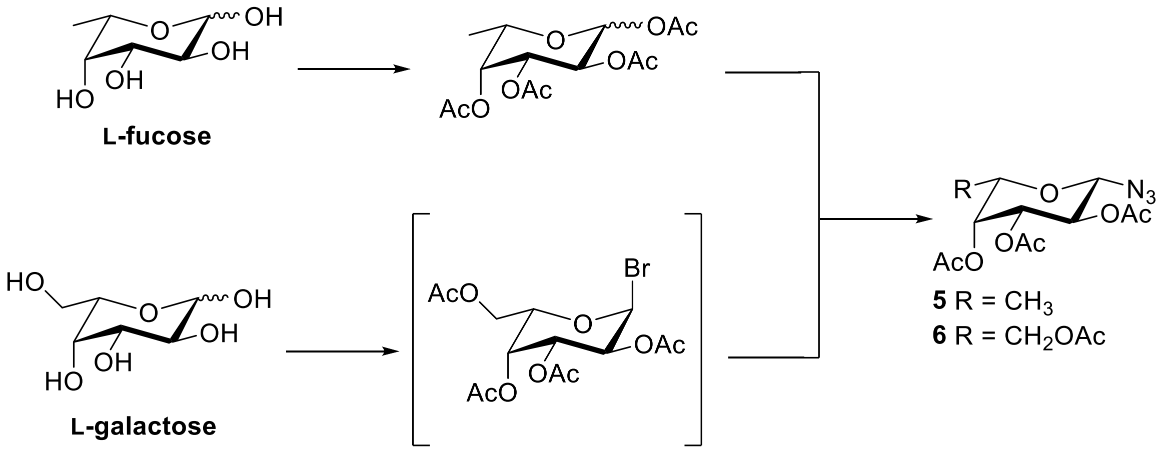

1.1. Synthesis of β-L-fucosyl azide (**5**)

1.1.1. Synthesis of 1,2,3,4-tetra-O-acetyl L-fucopyranoside

L-fucose (500 mg, 3.05 mmol, 1 eq) was dissolved in pyridine (2.88 mL) and cooled to 0 °C. Acetic anhydride (2.88 mL, 30.05 mmol, 10 eq) was slowly added and the reaction mixture was stirred at room temperature overnight. The solvent was removed under reduced pressure by coevaporation with toluene. The crude product was used for the next reaction without further purification. Y = quant., Rf (EtOAc/MeOH: 98:2) = 0.85. (mixture of α/β pyranoside 70:30 and traces of furanoside form). Characterization data in agreement with those reported in literature [1]. ^{1}H NMR (400 MHz, $CDCl_3$) α-anomer δ 6.33 (d, *J* = 2.6 Hz, 1H, H-1), 5.37 - 5.28 (mult., 3H, H-2 + H-3 + H-4), 4.26 (q, *J* = 6.6 Hz, 1H, H-5), 2.20 – 1.97 (s, 12H, OAc), 1.15 (d, *J* = 6.5 Hz, 3H, H-6); β -anomer δ = 5.67 (d, *J* = 8.3 Hz, 1H, H-1), 5.26 (d, *J* = 3.4 Hz, 1H, H-4), 5.06 (dd, *J* = 10.4, *J* = 3.4 Hz, 1H, H-3), 3.99-3.91 (m, 1H, H- 5), 2.20 – 1.97 (m, 12H, OAc), 1.22 (d, *J*= 6.4 Hz, 3H, H-6). Furanose, selected signals δ = 6.18, 5.51, 5.17, 4.21, 2.11, 2.08, 2.07, 2.06, 1.30.

1.1.2. Synthesis of (1,2,3,4-tri-O-acetyl β-L-fucopyranosyl) azide (5) [2]

1,2,3,4-Tetra-*O*-acetyl-L-fucopyranoside (270 mg, 0.81 mmol, 1 eq) was dissolved in anhydrous dichloromethane (4 mL, conc. = 0.2 M) and cooled to 0 °C under N_2 atmosphere. Trimethylsilyl azide ($TMSN_3$, 0.14 mL, 1.05 mmol, 1.3 eq) and $SnCl_4$ (0.05 mL, 0.41 mmol, 0.5 eq) were added to the solution, and the reaction mixture was stirred at room temperature for 3 h. The reaction was diluted with dichloromethane (2 mL) and washed with sat. solution of $NaHCO_3$ (4 mL) and water (4 mL), dried over anhydrous Na_2SO_4, filtered and concentred under reduced pressure. The residue was suspended in isopropyl ether (~ 2 mL) and the solid was filtered under vacuum, giving pure **5** in 66% yield. Rf (*n*-Hex/AcOEt 2:1) = 0.52. Characterization data in agreement with those reported in literature [2]. ^{1}H NMR (400 MHz, $CDCl_3$) δ 5.27 (dd, *J* = 3.3, *J* = 0.8 Hz, 1H, H-4), 5.14 (dd, *J* = 10.3, *J* = 8.7 Hz, 1H, H-2), 5.03 (dd, *J* = 10.3, *J* = 3.4 Hz, 1H, H-3), 4.58 (d, *J* = 8.7 Hz, 1H, H-1), 3.90 (dq, *J* = 1.2 Hz, *J* = 6.4 Hz, 1H, H-5), 2.19 (s, 3H, OAc), 2.09 (s, 3H, OAc), 1.99 (s, 3H, OAc), 1.25 (d, *J* = 6.4 Hz, 3H, H-6).

1.2. Synthesis of β-L-galactosyl azide (**6**)

***2,3,4,6-tetra-O-acetyl-β-L-galactopyranosyl azide* (6)**[3]

To a cold suspension (0°C) of L-galactose (100 mg, 0.56 mmol, 1 eq) in acetic anhydride (0.27 mL, 2.81 mmol, 5 eq), a first portion of HBr/AcOH (30%, 0.15 mL, 0.56 mmol , 1 eq) was slowly added and the reaction mixture was allowed to warm to room temperature and left to stir for 15 min (until a clear solution was obtained). The mixture was cooled to 0 °C and the remaining portion of HBr/AcOH (30 %, 0.30 mL, 1.12 mmol, 2 eq) was slowly added. The reaction mixture was stirred at room temperature until TLC showed full consumption of the starting materials (~ 2 hours), then it was coevaporated with toluene under reduced pressure. The crude was dissolved in dichloromethane (2.8 mL, conc = 0.2 M) and sodium azide (73 mg, 1.12 mmol, 2 eq), tetrabuthylammonium hydrogen sulfate (TBAHS, 29 mg, 0.084 mmol, 15% mol) and a 1M aqueous solution of Na_2CO_3 (3.92 mL, 7 eq) were added. The biphasic reaction mixture was vigorously stirred for additional 1.5 hours, then was diluted with dichloromethane (4 mL) and the two phases were separated. The organic layer was washed with water (2 x 5 mL), dried over anhydrous Na_2SO_4, filtered and concentred under vacuum. The product was further purified by automatic chromatography (SFAR 10 g, *n*-Hex/AcOEt 8:2), furnishing the desired compound as yellow oil. Y= 70%. Rf (*n*-Hex/AcOEt 1:1) = 0.55.^{1}H NMR (400 MHz, $CDCl_3$) δ 5.42 (d, *J* = 3.0 Hz, 1H, H-4), 5.16 (dd, *J* = 10.2, *J* = 8.8 Hz, 1H, H-2), 5.03 (dd, *J* = 10.3, *J* = 3.4 Hz, 1H, H-3), 4.59 (d, *J* = 8.7 Hz, 1H, H-1), 4.22 – 4.11 (m, 2H, H-6, H-6′), 4.01 (t, *J* = 6.5 Hz, 1H, H-5), 2.17 (s, 3H, OAc), 2.09 (s, 3H, OAc), 2.06 (s, 3H, OAc), 1.99 (s, 3H, OAc).

2. STD NMR

2.1 Competition experiments

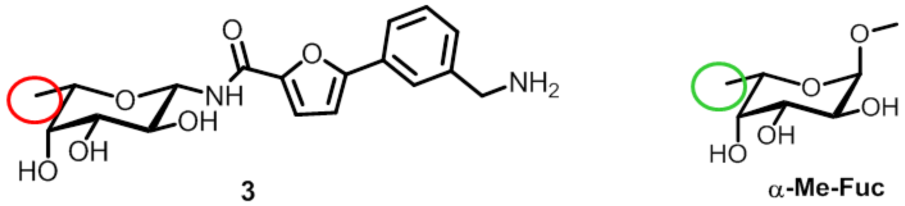

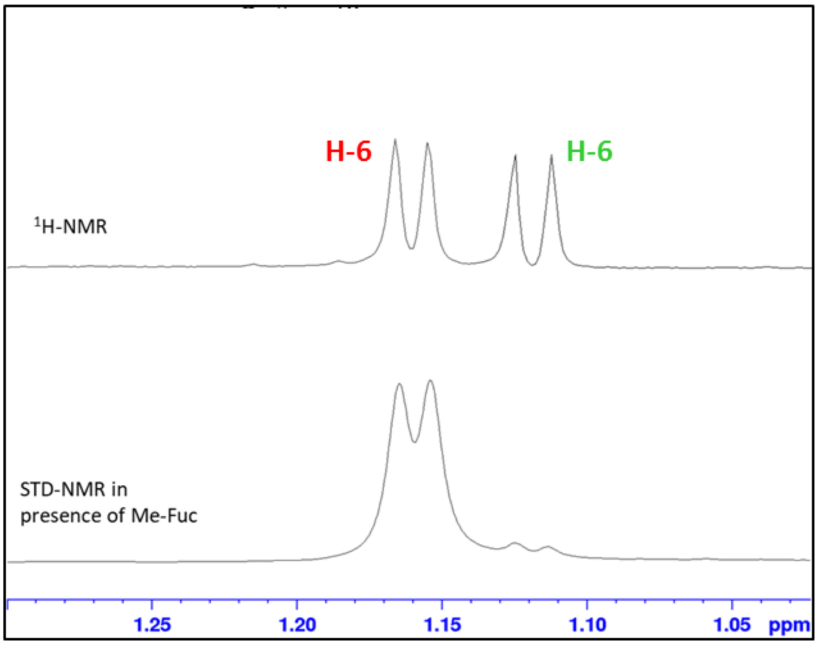

Figure S1. Competition experiment between **3** and α-methyl fucoside. ^{1}H (top) and STD NMR (bottom) of a 1:1 mixture of **3** and α-methylfucoside in the presence of BC2L-C-Nt. The doublets for the fucose methyl groups are at 1.17 ppm for **3** and 1.12 ppm for α-methylfucoside . The STD experiment was acquired on a Bruker Avance 600 MHz at 298 K, irradiating for 0.98 s at -0.05 ppm in phosphate buffer pH= 7.4.

- To a solution of **3** (2 mmol) and BC2L-C-Nt (2 μmol) in 500 μL of pH 7.4 buffered water solution, α-methyl fucoside (2 mmol) was added and the STD spectrum was recorded irradiating for 0.98 s at -0.05 ppm
- A 20% intensity decrease for the fucose moiety and 26% intensity decrease for the aromatic moiety of **3** (Table S1) was observed. The relative intensity of the C6 peaks of **3** (1.17 ppm) and α-methylfucoside (1.12 ppm) is shown in Figure S1.

2.2 Absolute and relative STD % for compounds **3** and **4**

Table S1. Absolute and relative STD % for compound **3** calculated from the STD NMR spectrum in the presence of BC2L-C-Nt (ligand:protein ratio 1000:1, on resonance -0.05 ppm). The absolute STD % calculated in the competition experiment with α-methylfucoside is also included (an absolute STD % of 0.5 % was calculated for the methyl group of α-methyl fucoside).

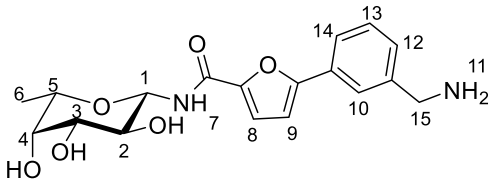

3

Atom	ppm	Absolute STD	Relative STD	Absolute STD after addition of α-methylfucoside
10	7.82	1.78 %	70 %	1.31 %
14	7.8	1.78 %	70 %	1.31 %
13	7.47	1.78 %	70 %	1.31 %
12	7.38	1.78 %	70 %	1.31 %
8	7.29	0.7 %	30 %	0.4 %
9	6.93	0.18 %	7 %	0.16 %
1	5.02	nd	nd	0.4 %
15	4.15	0.5 %	20 %	0.4 %
5	3.85	0.5 %	20 %	nd
2-4	3.74-3.66	0.5 %	20 %	nd
6	1.17	2.5 %	100 %	2.05 %

Table S2. Absolute and relative STD % for compound **4** calculated from STD NMR spectrum in presence of BC2L-C-Nt (1000:1 ligand:protein ratio, on resonance -0.05 ppm). The absolute STD % calculated in the competition experiment with α-methylfucoside is also included (an absolute STD % of 0.8 % was calculated for the methyl group of α-methyl fucoside).

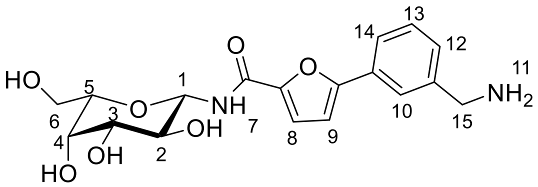

4

Atom	ppm	Absolute STD	Relative STD	Absolute STD after addition of fucose
10	7.82	1.66 %	100 %	0.65 %
14	7.8	1.66 %	100 %	0.65 %
13	7.47	1.66 %	100 %	0.65 %
12	7.37	1.66 %	100 %	0.65 %
8	7.31	1.05 %	63 %	0.46 %
9	6.94	0.32%	19 %	0.26 %
1	5.04	nd	nd	nd
15	4.14	0.52 %	31 %	0.51 %
5	3.93	0.3 %	18 %	nd
2, 3, 4, 6	3.77-3.66	0.3 %	18 %	nd

3. Isothermal titration calorimetry

Figure S2. ITC titration of BC2L-C-Nt by **3** (stoichiometry fixed to 1). Thermograms are represented on the top and fitted curve at the bottom for a duplicate (left and central panel). As the baseline in the thermogram indicated buffer mismatch, a blank experiment was performed by injecting the ligand into the buffer solution. The signal obtained (right panel) was then subtracted in the fitting step to take into account the mismatch.

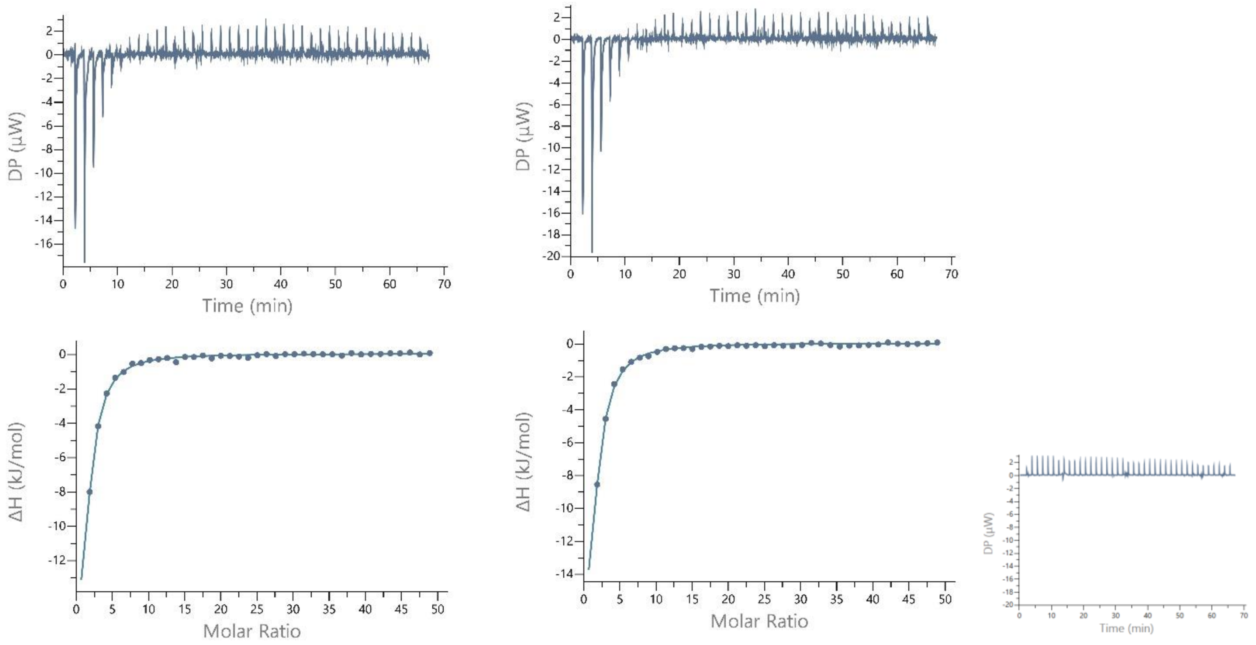

Figure S3. ITC titration of BC2L-C-Nt by **4** (stoichiometry fixed to 1). Thermograms are represented on the top and fitted curve at the bottom for a duplicate (left and central panel). As the baseline in the thermogram indicated buffer mismatch, a blank experiment was performed by injecting the ligand into the buffer solution. The signal obtained (right panel) was then subtracted in the fitting step to take into account the mismatch.

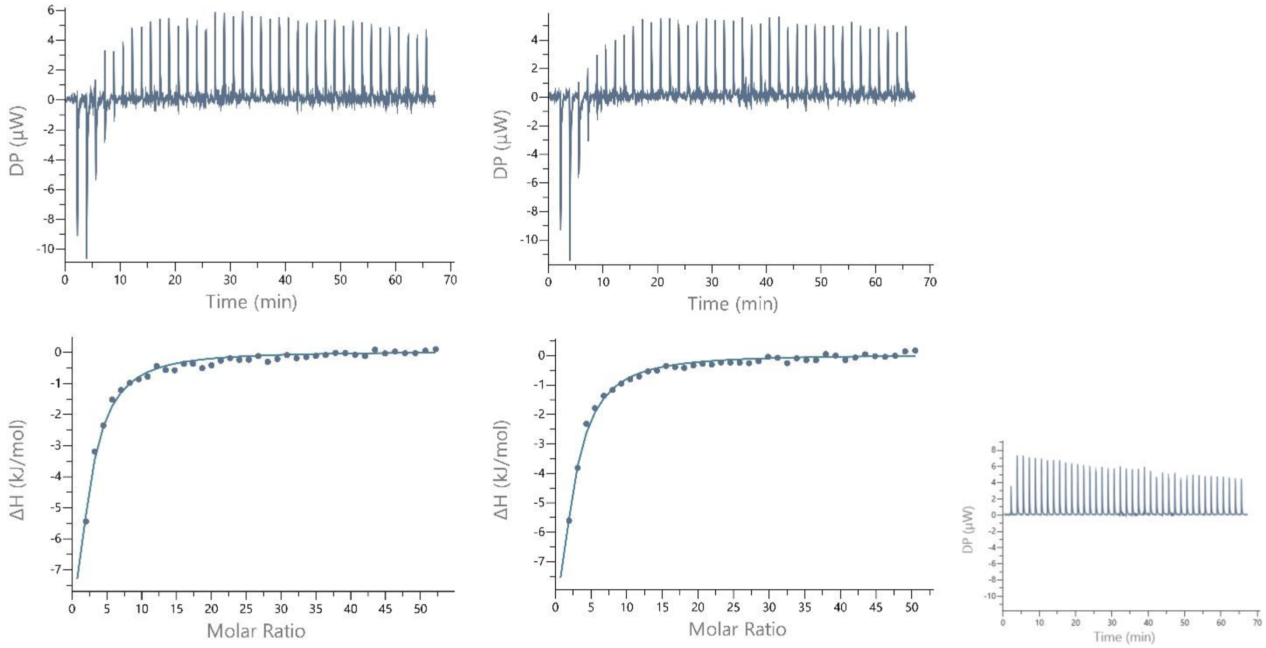

Figure S4. ITC titration of BC2L-C-Nt by **17** (stoichiometry fixed to 1). Thermograms are represented on the top and fitted curve at the bottom for a duplicate (left and central panel). As the baseline in the thermogram indicated buffer mismatch, a blank experiment was performed by injecting the ligand into the buffer solution. The signal obtained (right panel) was then subtracted in the fitting step to take into account the mismatch.

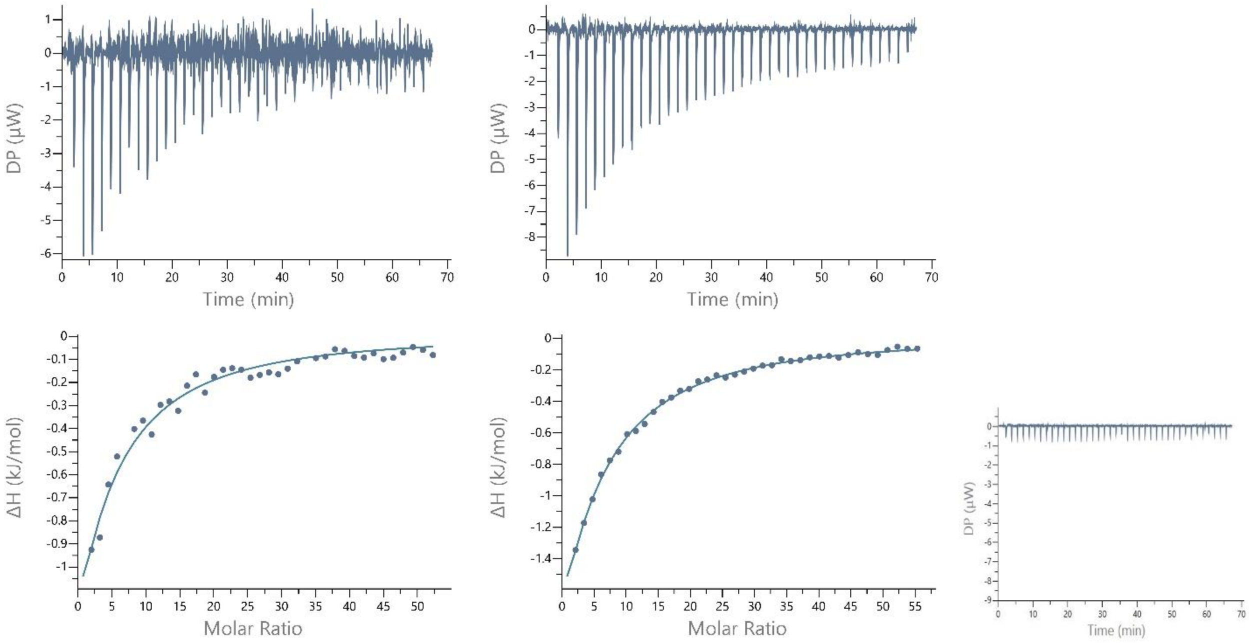

4. Comparison of X-ray and docked structure

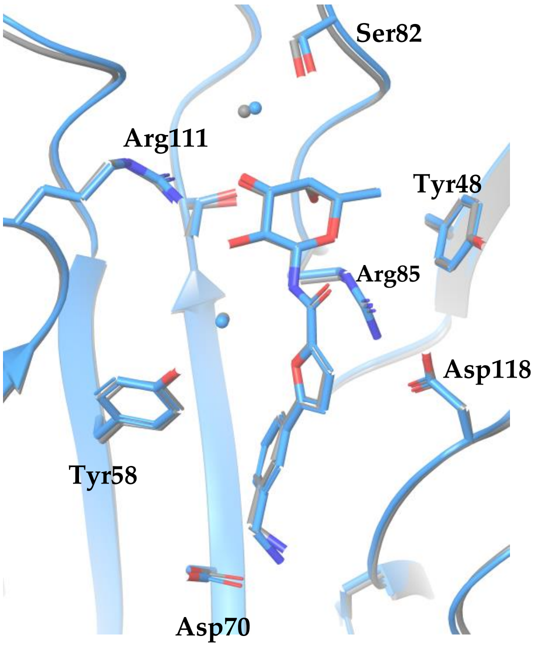

Figure S5. Overlay of the best pose of **3** obtained by docking (light blue, GlideScore: -10.0 kcal/mol with the XP scoring function) with the X-ray structure of the BC2L-C-Nt complex (grey). Water molecules are depicted as spheres.

5. NMR spectra of new compounds

Methyl 5-(3-((tert-butoxycarbonyl)aminomethyl)phenyl)furan-2-carboxylate (11)

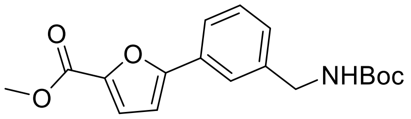

^{1}H NMR (400 MHz, $CDCl_3$)

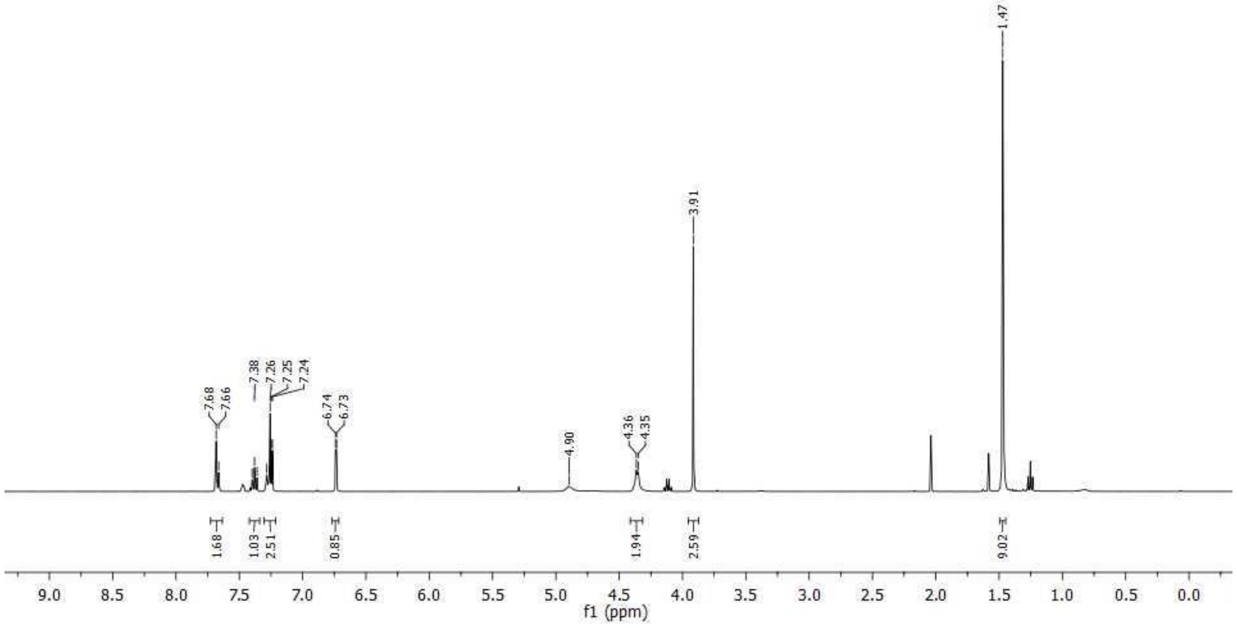

HSQC (400 MHz, $CDCl_3$)

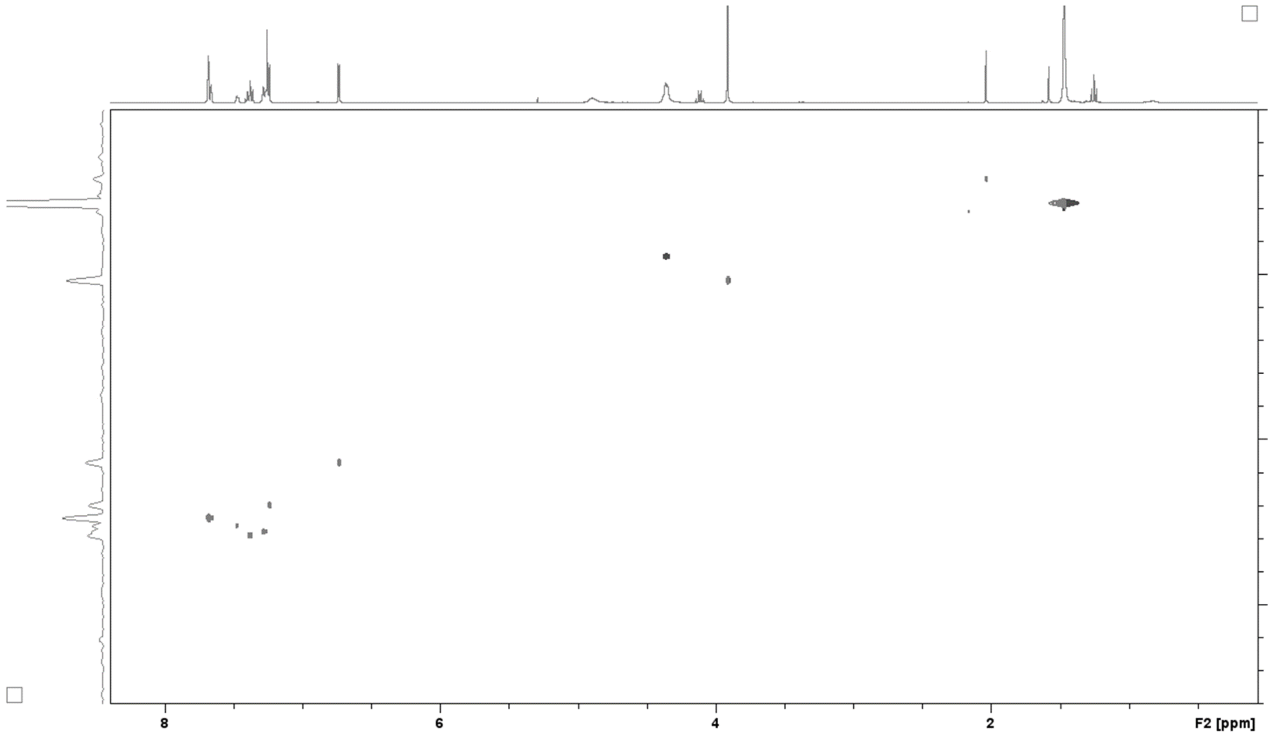

5-(3-((tert-butoxycarbonyl)aminomethyl)phenyl)furan-2-carboxylic acid (12)

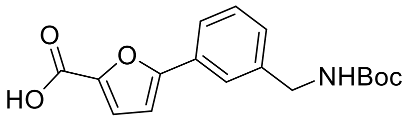

^{1}H NMR (400 MHz, $CDCl_3$)

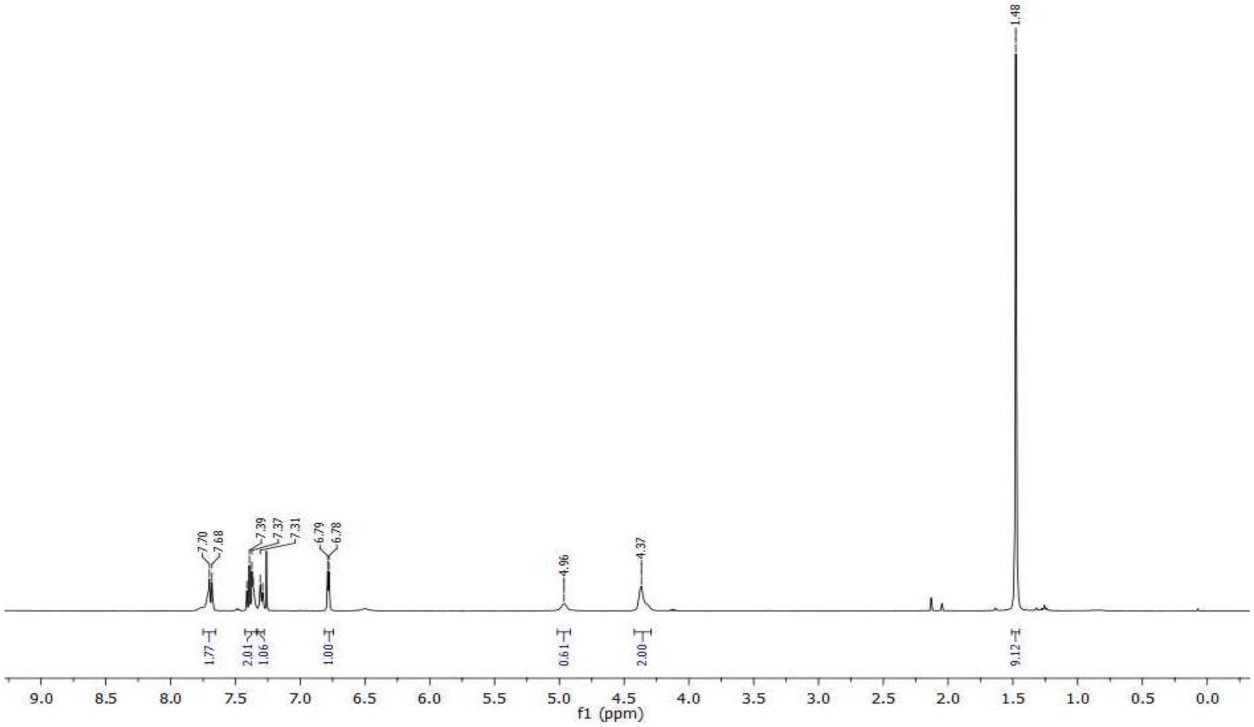

HSQC (400 MHz, $CDCl_3$)

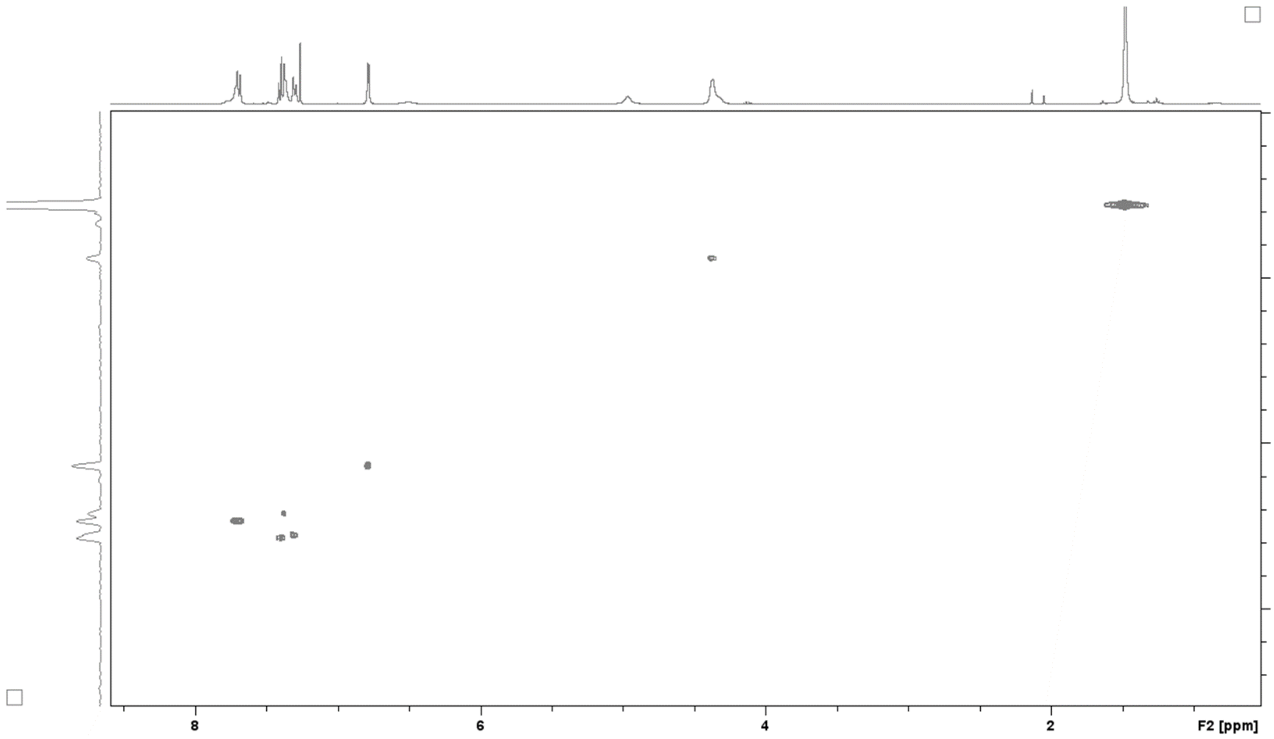

(5-(3-((tert-butoxycarbonyl)aminomethyl)phenyl)furan-2-carboxamido)-2,3,4-tri-O-acetyl-β-L-fucopyranose (13)

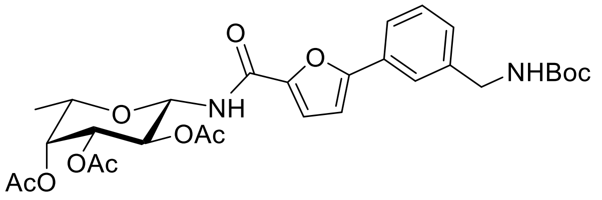

^{1}H NMR (400 MHz, $CDCl_3$)

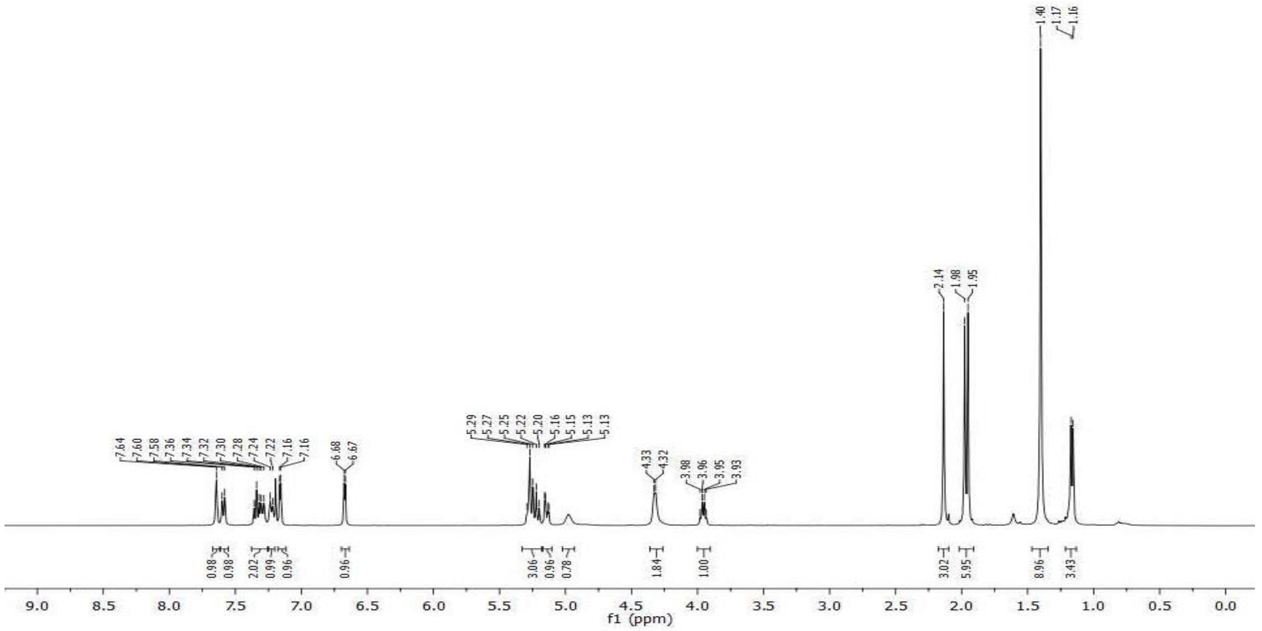

HSQC (400 MHz, $CDCl_3$)

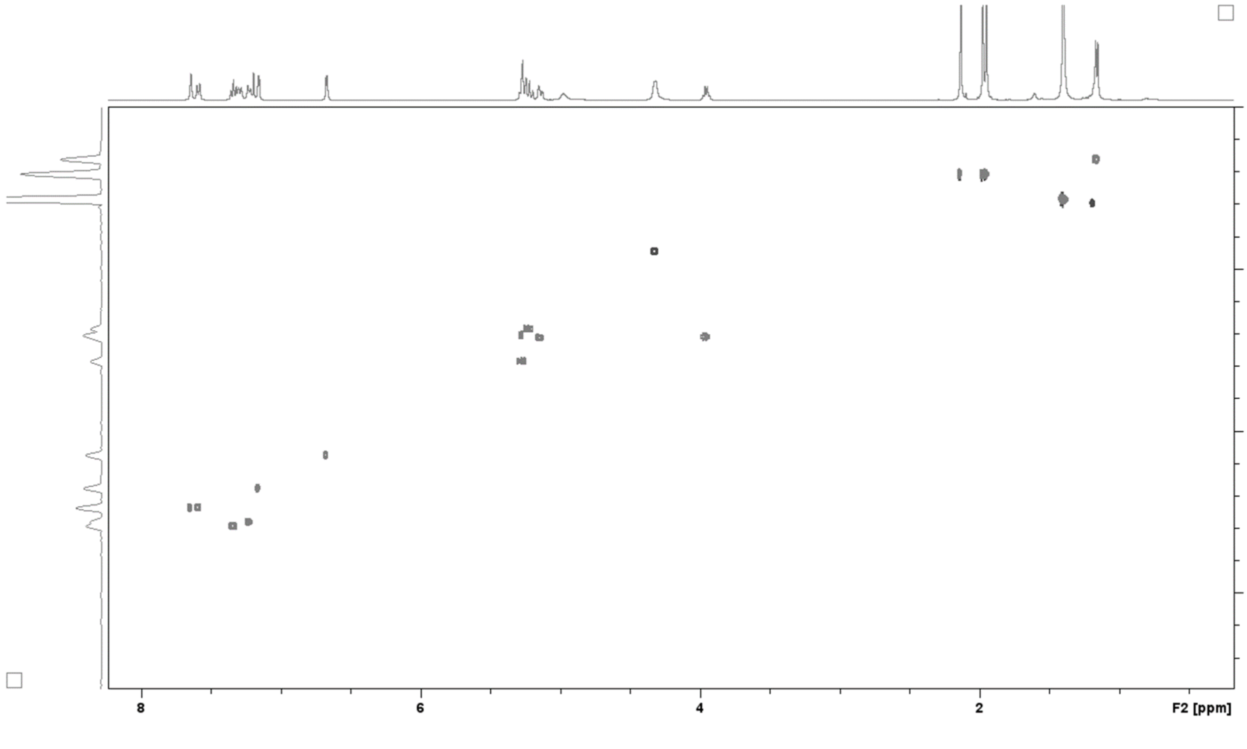

(5-(3-((tert-butoxycarbonyl)aminomethyl)phenyl)furan-2-carboxamido)-2,3,4,6-tetra-O-acetyl-β-L-galactopyranose (14)

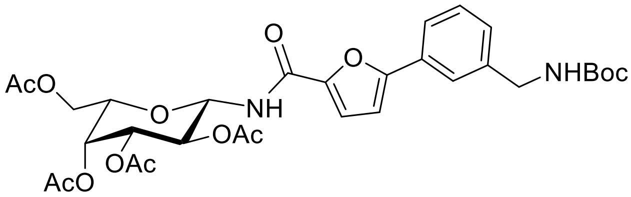

^{1}H NMR (400 MHz, $CDCl_3$)

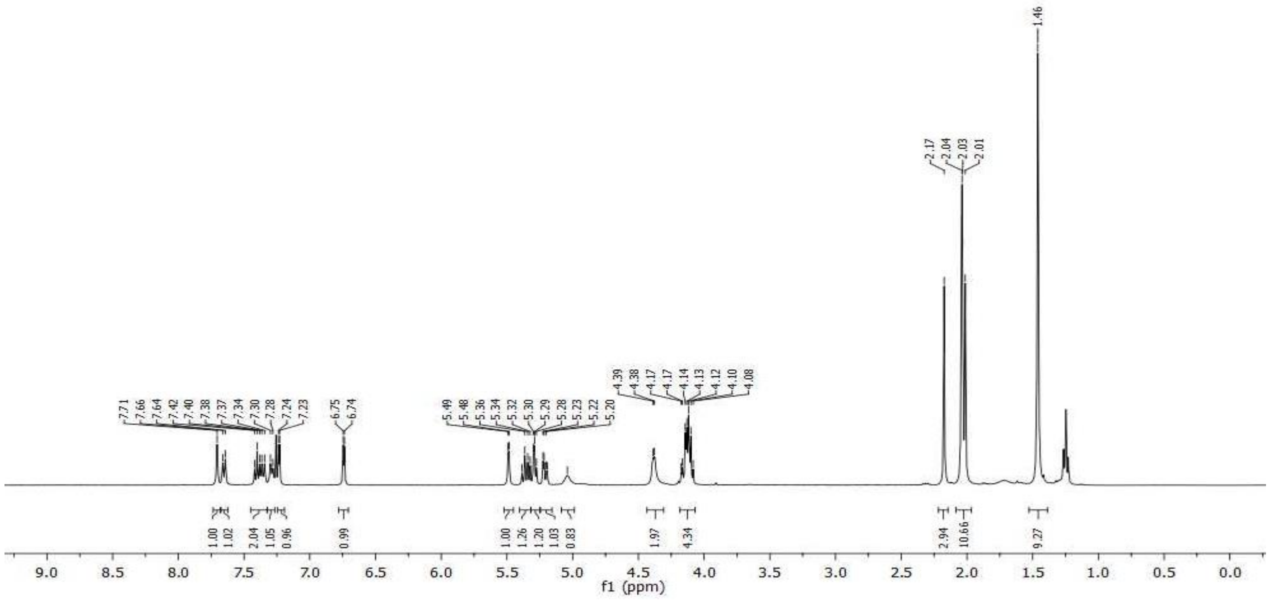

HSQC (400 MHz, $CDCl_3$)

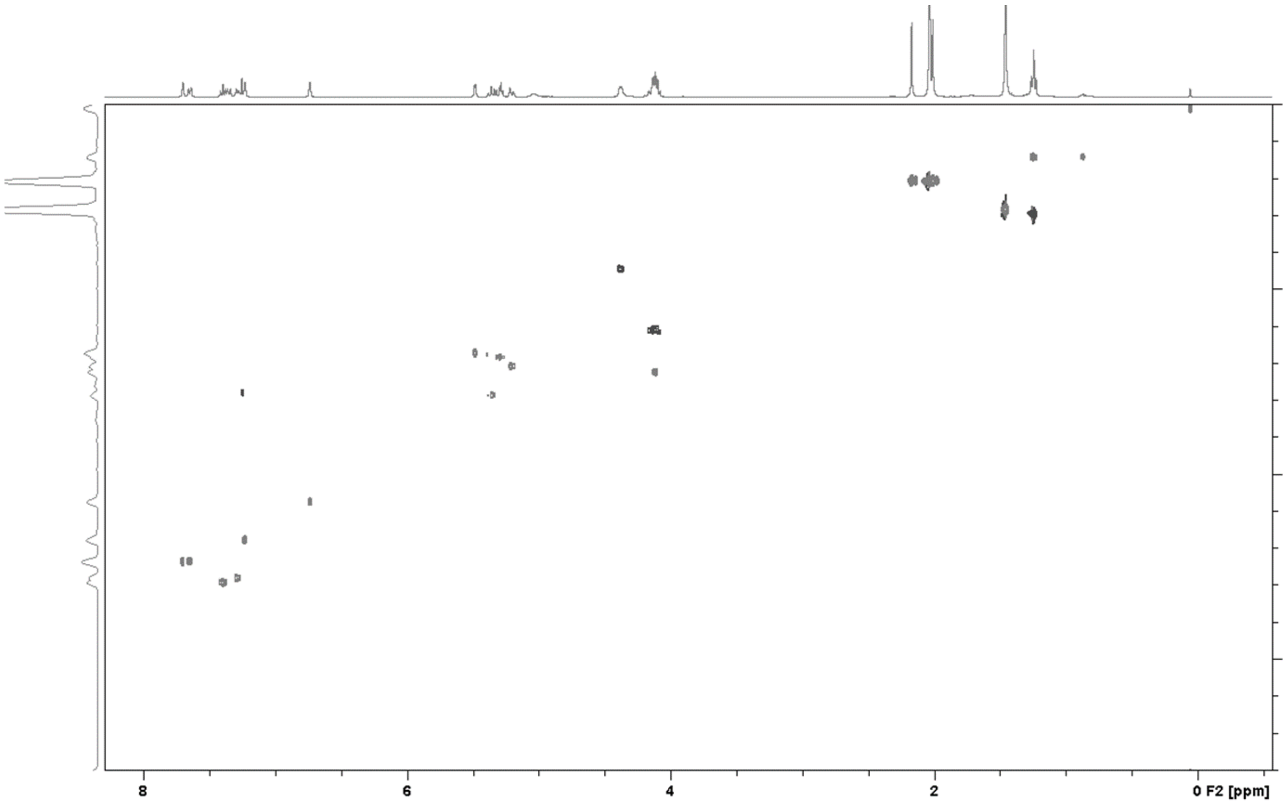

(5-(3-((tert-butoxycarbonyl)aminomethyl)phenyl)furan-2-carboxamido)-β-L-fucopyranose

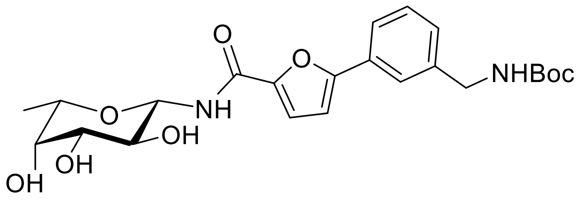

^{1}H NMR (400 MHz, CD_3OD)

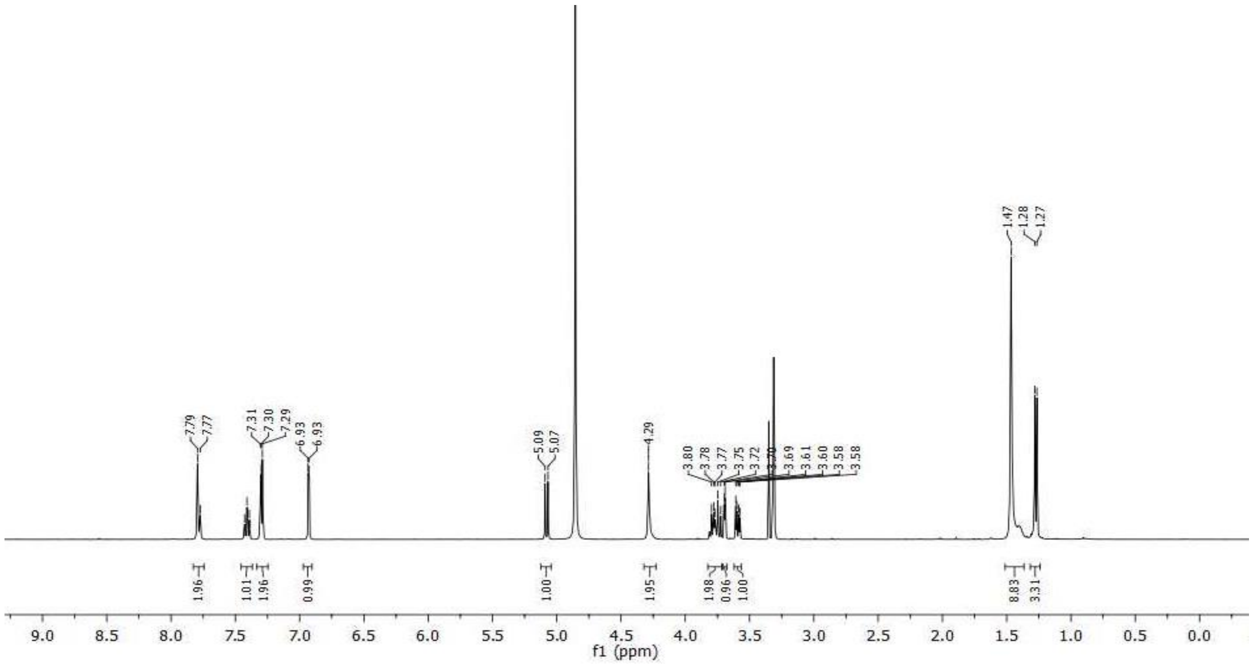

HSQC (400 MHz, CD_3OD)

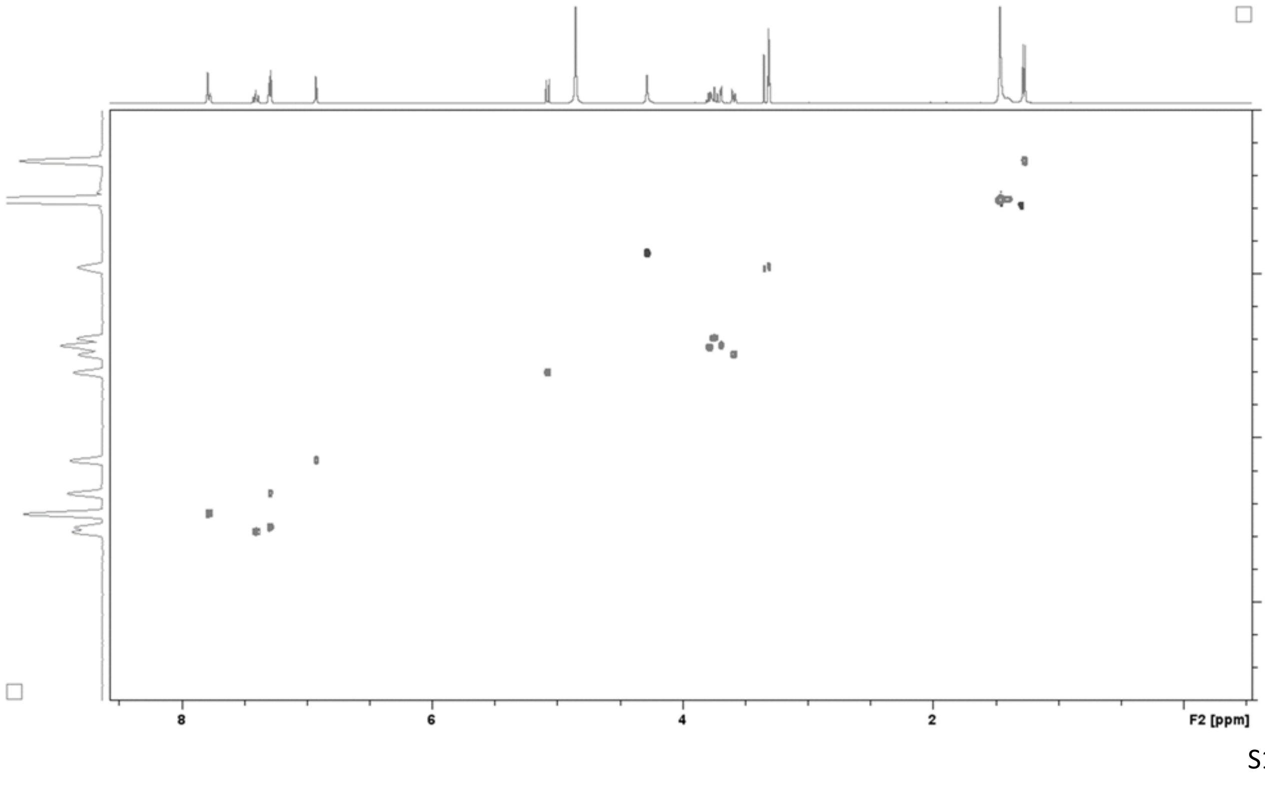

(5-(3-((tert-butoxycarbonyl)aminomethyl)phenyl)furan-2-carboxamido)-β-L-galactopyranose

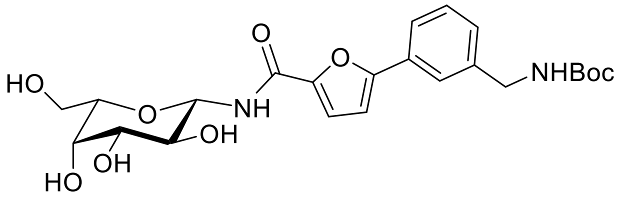

^{1}H NMR (400 MHz, CD_3OD)

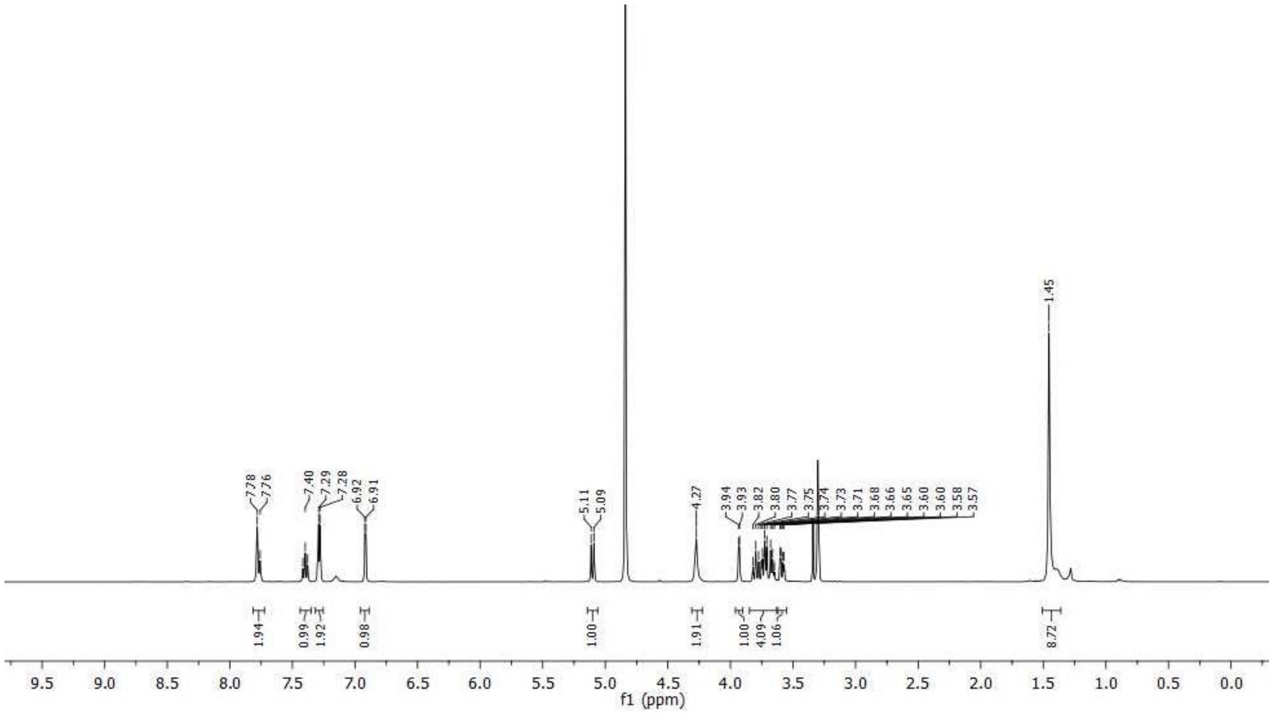

HSQC (400 MHz, CD_3OD)

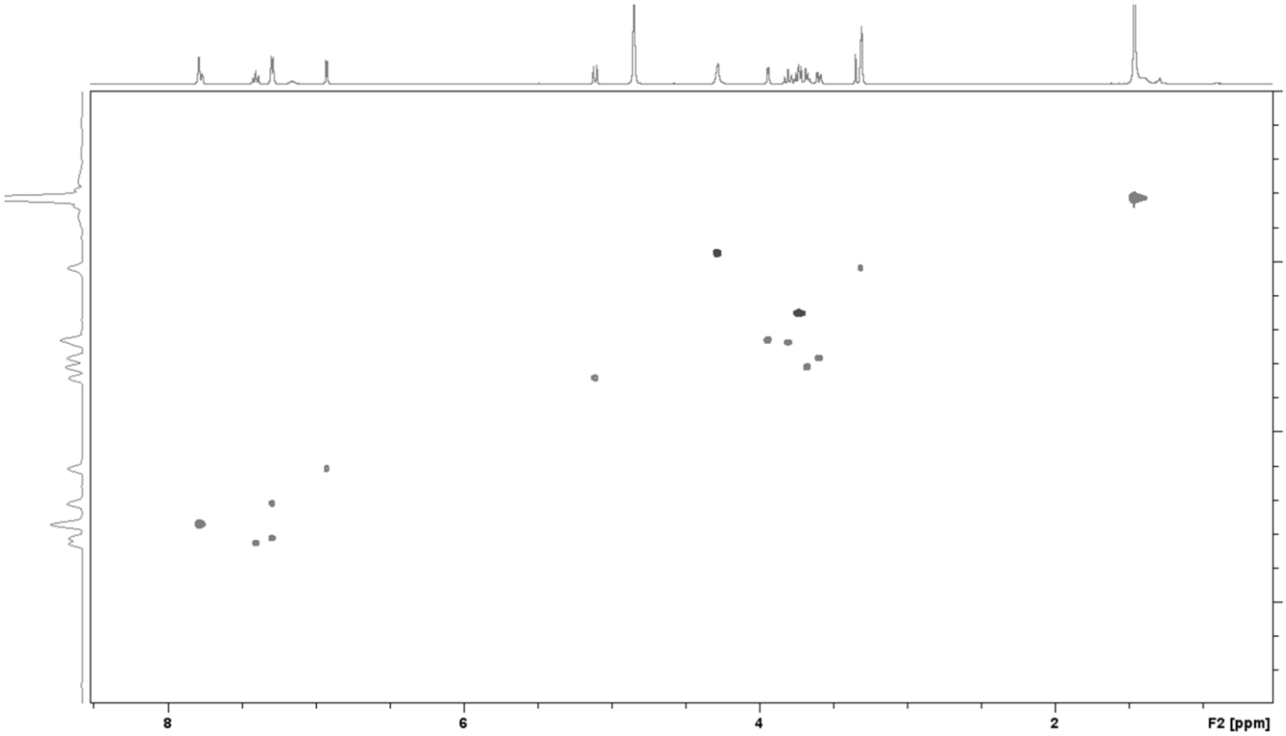

(5-(3-aminomethyl)phenyl)furan-2-carboxamido)-β-L-fucopyranose (3)

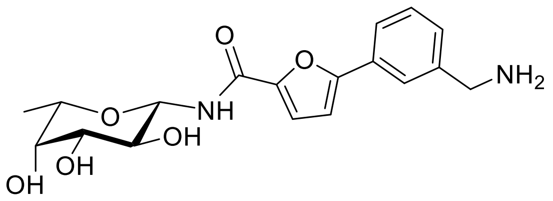

^{1}H NMR (400 MHz, CD3OD)

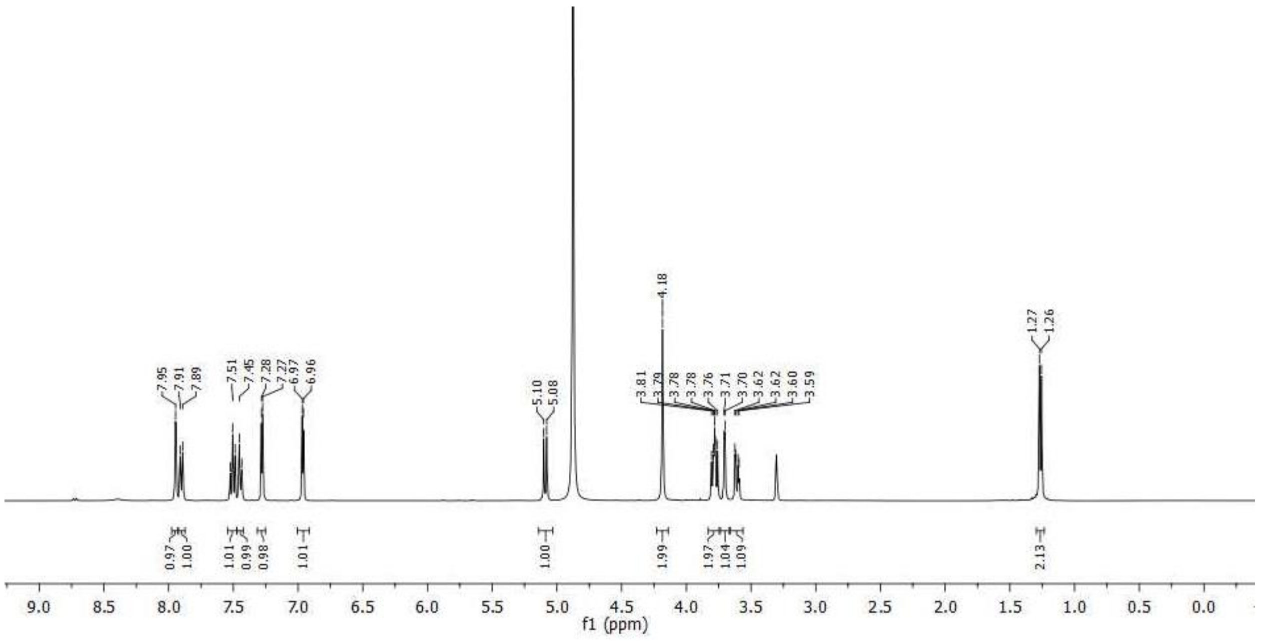

^{13}C NMR (100 MHz, CD3OD)

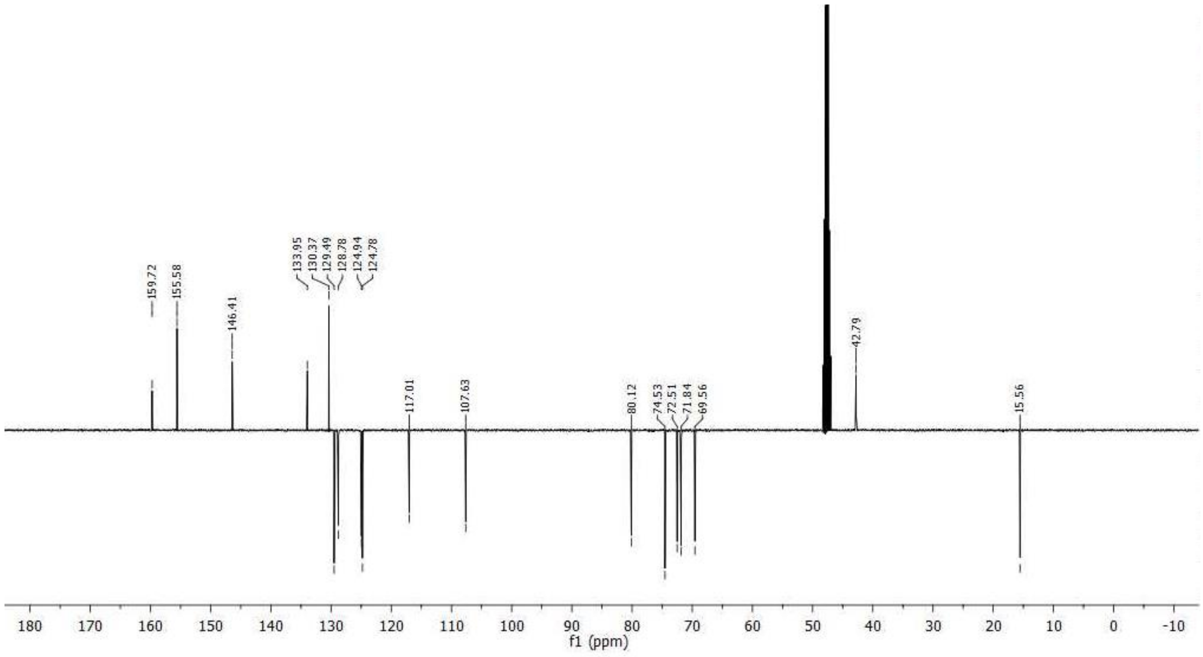

(5-(3-aminomethyl)phenyl)furan-2-carboxamido)-β-L-galactopyranose (4)

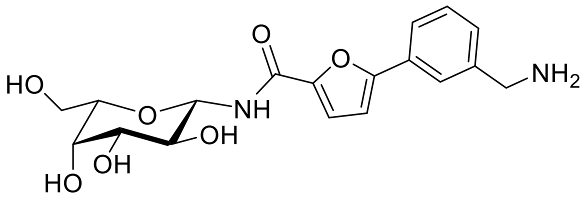

^{1}H NMR (400 MHz, CD3OD)

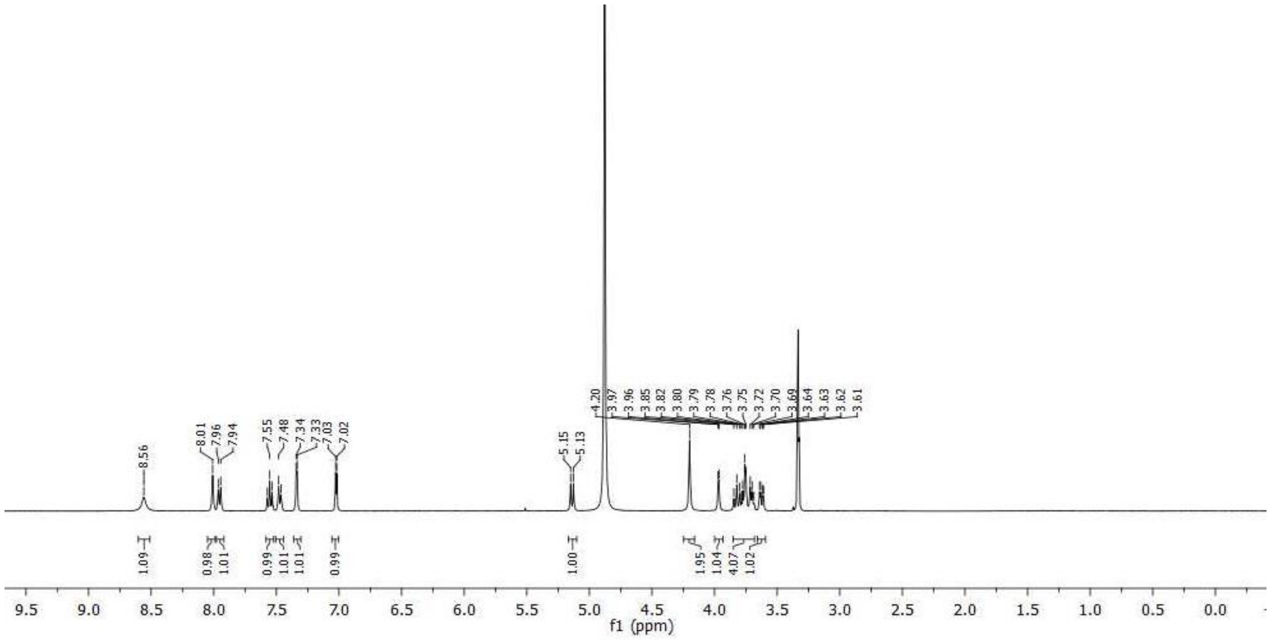

^{13}C NMR (100 MHz, CD3OD)

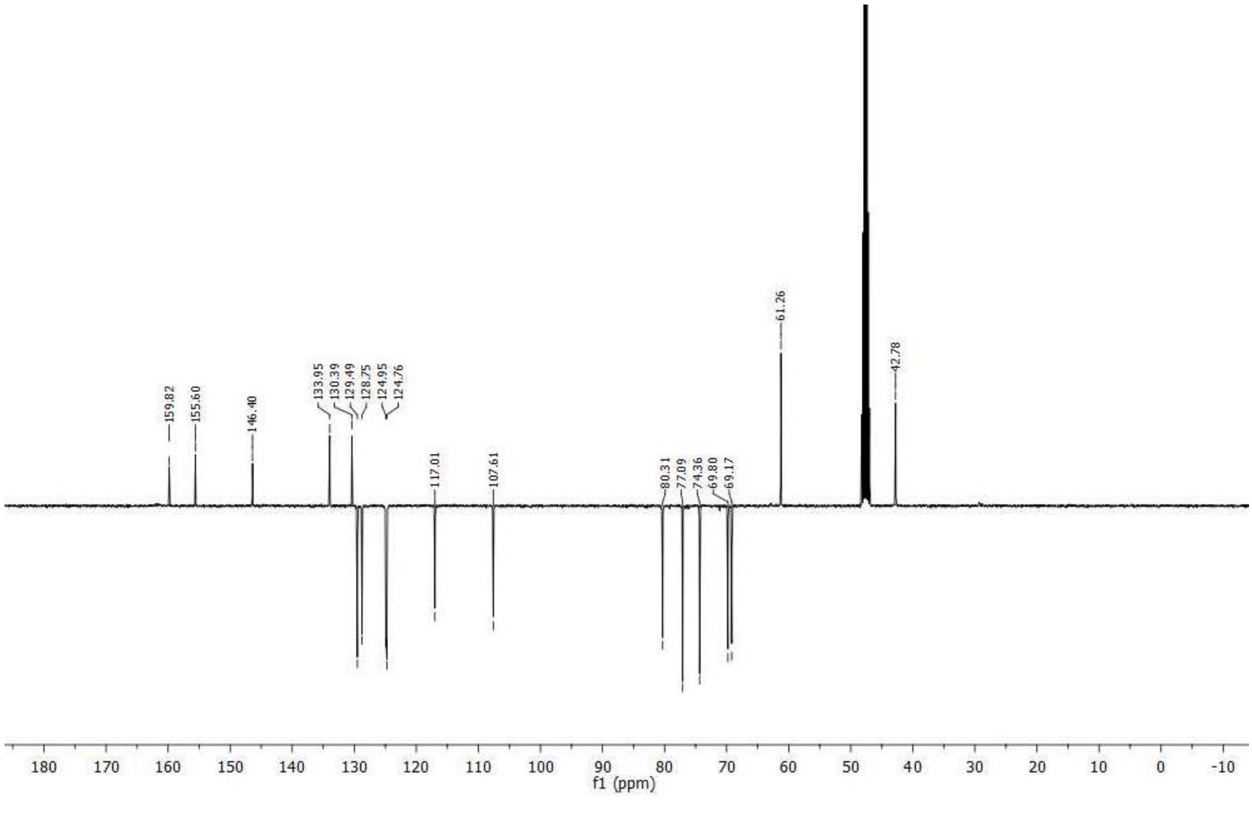

References

1. Duléry, V.; Renaudet, O.; Philouze, C.; Dumy, P. α and β l-Fucopyranosyl oxyamines: key intermediates for the preparation of fucose-containing glycoconjugates by oxime ligation. *Carbohydr. Res.* **2007**, *342*, 894–900, doi:10.1016/j.carres.2007.02.003.

2. Palomo, C.; Aizpurua, J.M.; Balentová, E.; Azcune, I.; Santos, J.I.; Jiménez-Barbero, J.; Cañada, J.; Miranda, J.I. "Click" saccharide/β-lactam hybrids for lectin inhibition. *Org. Lett.* **2008**, *10*, 2227–2230, doi:10.1021/ol8006259.

3. Kumar, R.; Tiwari, P.; Maulik, P.R.; Misra, A.K. A Generalized Procedure for the One-Pot Preparation of Glycosyl Azides and Thioglycosides Directly from Unprotected Reducing Sugars under Phase-Transfer Reaction Conditions. *European J. Org. Chem.* **2005**, *2006*, 74–79, doi:https://doi.org/10.1002/ejoc.200500646.
